# Nanoscale organization of two-dimensional multimeric pMHC reagents with DNA origami for CD8^+^ T cell detection

**DOI:** 10.1038/s41467-022-31684-8

**Published:** 2022-07-07

**Authors:** Yueyang Sun, Lu Yan, Jiajia Sun, Mingshu Xiao, Wei Lai, Guangqi Song, Li Li, Chunhai Fan, Hao Pei

**Affiliations:** 1 Shanghai Key Laboratory of Green Chemistry and Chemical Processes, School of Chemistry and Molecular Engineering, East China Normal University, 500 Dongchuan Road, 200241 Shanghai, China; 2 Department of Gastroenterology and Hepatology, Zhongshan Hospital of Fudan University, Shanghai, China; 3 School of Chemistry and Chemical Engineering, and Institute of Molecular Medicine, Renji Hospital, School of Medicine, Shanghai Jiao Tong University, 200240 Shanghai, China; 4 Institute of Eco-Chongming, 202162 Shanghai, China

**Keywords:** Molecular self-assembly, Lymphocyte activation, T cells, Nanoscale biophysics, Assay systems

## Abstract

Peptide-MHC (pMHC) multimers have excelled in the detection of antigen-specific T cells and have allowed phenotypic analysis using other reagents, but their use for detection of low-affinity T cells remains a challenge. Here we develop a multimeric T cell identifying reagent platform using two-dimensional DNA origami scaffolds to spatially organize pMHCs (termed as dorimers) with nanoscale control. We show that these dorimers enhance the binding avidity for low-affinity antigen-specific T cell receptors (TCRs). The dorimers are able to detect more antigen-specific T cells in mouse CD8^+^ T cells and early-stage CD4^+^CD8^+^ double-positive thymocytes that express less dense TCRs, compared with the equivalent tetramers and dextramers. Moreover, we demonstrate dorimer function in the analysis of autoimmune CD8^**+**^ T cells that express low-affinity TCRs, which are difficult to detect using tetramers. We anticipate that dorimers could contribute to the investigation of antigen-specific T cells in immune T cell function or immunotherapy applications.

## Abstract (web summary)

MHC-peptide multimers are important reagents for detecting antigen specific T cells. Here the authors show that DNA scaffolds can be used to make MHC-peptide multimers and the avidity controlled so that low abundance or T cells with low affinity TCR can be detected using these reagents.

## Introduction

T cells have an important function to eliminate pathogens and surveil pathological cells in adaptive immunity^1, 2^. They discriminate between self- and non-self-antigens through the recognition of peptide–major histocompatibility complexes (pMHC) by surface T cell receptors (TCRs)^3–7^. Phenotypic-profiling antigen-specific T cells allows to inform disease management (e.g., disease development, disease diagnosis, therapeutic efficacy assessment) and guides the development of immunotherapy strategies^8–11^. The pMHC multimer technology that relies on the multivalent presentation of pMHC to increase the avidity of TCR binding^12–15^, has received extensive attention. Various pMHC multimers have been developed through increasing the stoichiometry of pMHC molecules (e.g., tetramer, octamer, dextramer) or controlling the orientation of pMHC molecules (e.g., pentamer)^16–21^. However, it remains challenging to accurately and sensitively analyze rare and/or low-affinity T cells, which is mainly due to insufficient binding avidity using low-valency reagents or nonspecific staining using higher valency reagents.

DNA origami has proven its utility in precise organization of objects (e.g., small molecules, proteins, nucleic acids, and nanoparticles) on the nanoscale^22–27^, owing to its high programmability and precise addressability^28–31^. Here we report DNA-origami-based pMHC multimers (termed as dorimers) with precise control of spacing and valency for antigen-specific T cells detection. We demonstrate that dorimers with relatively shorter pMHC spacing and higher pMHC valency show enhanced avidity of TCR binding. The dorimers are able to detect more antigen-specific T cells in mouse CD8^+^ T cells and early-stage CD4^+^CD8^+^ double-positive thymocytes that express 10- to 30-fold less dense TCRs, compared with the equivalent tetramers and dextramers. The dorimers are also applicable for detecting autoimmune CD8^+^ T cells that express low-affinity TCRs, which are difficult to detect using tetramers.

## Results

### 2D pMHC multimers with nanoscale organization

**Fig. 1:**
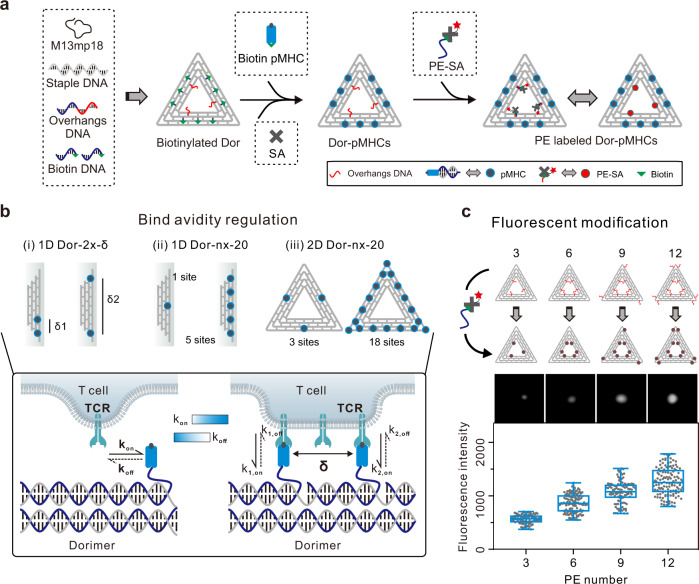
Nanoscale organization of pMHC molecules with DNA origami. **a** Schematic illustrating the construction of 2D pMHC multimers with triangular DNA origami scaffolds. Single-stranded M13 phage genomic DNA was mixed with predesigned staple strands (including overhangs-DNA, and biotin-DNA) at the ratio of 1:10 for assembly of the biotinylated DNA origami, where biotins served as SA-binding sites. SA molecules and biotinylated pMHC molecules were successively assembled onto DNA origami based on the biotin–streptavidin interaction, generating Dor-pMHCs. Three pMHC molecules are assembled at each SA-binding site. Poly-A Oligonucleotide-conjugated PE-SA was site-specifically coupled to Dor-pMHCs through hybridization to overhangs-DNA (poly-T oligonucleotides, labeled by red) extended from predesigned locations. **b** Schematic overview of the determinants that potentially regulate avidity of TCR binding, including (i) the inter-pMHC spacing (δ: 20–80 nm), (ii) 1D (*n*: 1–5 binding sites) and (iii) 2D (*n*: 3–18 binding sites) with different pMHC stoichiometry. The constructed Dor-pMHCs were abbreviated as Dor-*nx*-*δ* (*n* indicates the number of SA-binding site, *δ* indicates the inter-pMHC spacing on each edge). **c** The fluorescence intensity of DNA origami labeled with different numbers of PE proteins (ranging from 3 to 12) and their corresponding fluorescence images. Red dots indicated PE proteins assembled on DNA origami. Data represent the mean ± largest/smallest. (Data were collected based on about 100 counts of samples: 3, *n* = 96 particles; 6, *n* = 133 particles; 9, *n* = 105 particles; 12, *n* = 126 particles). Source data are provided as a Source Data file. Dor DNA origami, SA streptavidin, pMHC peptide–major histocompatibility complexes, PE-SA phycoerythrin-labeled streptavidin.

We fabricated triangular DNA origamis (edge length ∼120 nm) as 2D scaffolds (Fig. 1a, Supplementary Data 1)^32^, mimicking the mode of cross-junctional binding of T cell to antigen-presenting cell^33–35^. To build DNA-origami-based multimeric pMHCs (Dor-pMHCs), biotinylated DNA origami (Dor) was firstly assembled by labeling specific DNA staple strands with biotins (Fig. 1a, left). The biotin molecules extended from the same side of DNA origamis serve as streptavidin (SA)-binding sites, which could enable pMHC molecules to face the same direction to facilitate the interaction with TCRs. Next, the commercially available SA molecules were introduced as linkages into the system, enabling the direct attachment of biotinylated pMHC monomers to SA-binding sites based on the biotin–streptavidin interaction (Fig. 1a)^36^. Note that three pMHC molecules were assembled at each SA-binding site. Then, phycoerythrin (one type of fluorescence protein)-labeled SA (denoted as PE-SA) was site-specifically coupled to the DNA origami, through hybridizing to overhang strands that are extended from predesigned locations (Fig. 1a).^37–39^ Using this approach, we can present the SA-binding sites varying from 1 to 18 in variable nanoscale spatial organizations (corresponding to the pMHC valency varying from 3 to 54), enabling precise control over pMHC stoichiometry and inter-pMHC spacing (Fig. 1b).

Each step of the Dor-pMHCs assembly was characterized with atomic force microscopy (AFM) and agarose gel electrophoresis (AGE) to verify the validity of our design (Supplementary Figs. 1–6). AFM imaging showed that pMHC molecules were placed in designated locations on the top of the DNA origami scaffold, which is in line with the increased cross-section height after the successive assembly of SA and pMHC molecules onto the DNA origami (Supplementary Fig. 5). Moreover, the formation of PE-labeled Dor-pMHCs was confirmed by AGE analysis (Supplementary Fig. 6). The flow cytometry analysis was conducted to characterize the fluorescence intensity of DNA origami scaffolds labeled with different numbers of PE molecules varying from 3 to 12. The results showed that the fluorescence intensity was positively correlated with the number of PE molecules (Fig. 1c). Each Dor-pMHCs was labeled with three PE molecules for further study. Then, the stability and biocompatibility of Dor-pMHCs were assessed. After 72 h incubation, AGE analysis showed no obvious degradation of Dor-pMHCs (Supplementary Fig. 7), implying their robust structural integrity. Both the MTT assay and the Annexin V-FITC/PI double staining showed almost no cytotoxicity of Dor-pMHCs to spleen cells (Supplementary Figs. 8 and 9), indicating their biocompatibility.

### Effect of valency and inter-spacing of pMHC molecules on binding avidity of Dor-pMHCs

**Fig. 2:**
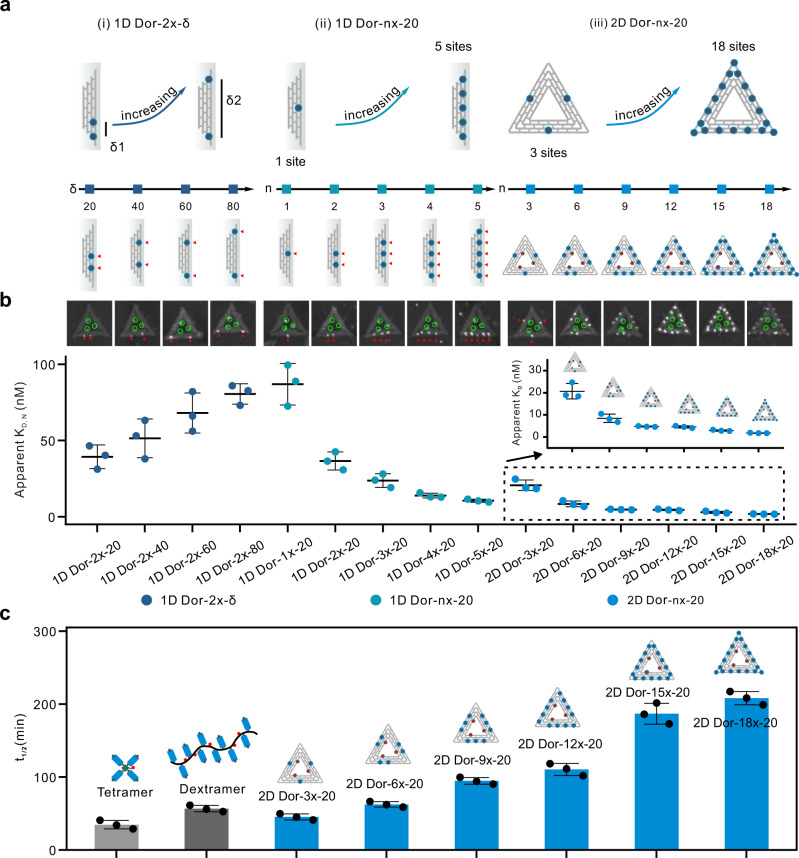
Effect of inter-pMHC spacing and pMHC valency on binding avidity of Dor-pMHCs binding to TCRs. **a** Schematic illustrating the design of three series of Dor-pMHCs, including 1D Dor-2*x*-*δ*, 1D Dor-*nx*-20, 2D Dor-*nx*-20. *n* indicates the number of SA-binding site and *δ* indicates the inter-pMHC spacing on each edge. **b** The apparent dissociation constant (apparent *K*_D,N_) of different types of Dor-pMHCs binding to OT-1 naive T cells at 4 °C. Inset: AFM images of Dor-pMHCs. *N* indicates the copy number of pMHC molecules on Dor-pMHCs. **c** Summary of the half-lives (*t*_1/2_) and dissociation kinetics of pMHC multimers (including tetramer, dextramer, and 2D Dor-*nx*-20) binding to OT-1 naive T cells at 4 °C measured using flow cytometry in the presence of saturated amounts of anti-H-2K^b^ MHC antibody at 4 °C. Data represent the mean ± s.d. from *n* = 3 (**b**, **c**) independent experiments. Source data are provided as a Source Data file.

We investigated the independent functions of inter-pMHC spacing versus pMHC copy number on the binding avidity of Dor-pMHCs to OT-1 naive T cells. Dor-pMHCs that present different pMHC copy number or different inter-pMHC spacing (denoted as Dor-*nx*-*δ*, *n* indicates the number of SA-binding site, and *δ* indicates the inter-pMHC spacing on each edge. Here *x* is used to distinguish the number of SA-binding site from the inter-pMHC spacing, since both are represented numerically.) were designed (Fig. 2a). Firstly, we investigated the impact of inter-pMHC spacing on the binding avidity of Dor-pMHCs to TCRs (Fig. 2a, left), using one series of Dor-pMHCs presenting two SA-binding sites with variable inter-pMHC spacings on the same edge (denoted as 1D Dor-2*x*-*δ*, *δ* ranging from 20 to 80 nm, Fig. 2b and Supplementary Fig. 10). The binding avidity of 1D Dor-2*x*-*δ* (containing specific OVA_257–264_ peptide, H-2K^b^) to ovalbumin (OVA)-specific CD8^**+**^ T cells was measured by monitoring the mean fluorescence intensity (i.e., PE) on T cells with flow cytometry. The binding avidity of 1D Dor-2*x*-*δ* was observed to be weaker with increasing the pMHC spacing, and their apparent dissociation constants (*K*_D,N_) increased from 39.4 to 80.5 nM (Fig. 2b and Supplementary Fig. 11a), indicating that relatively short pMHC spacing facilitated pMHC multimer-TCR binding. Considering three pMHC molecules assembled at each SA-binding site, further decreasing the spacing may hamper binding due to the steric hindrance. Hence, *δ* = 20 nm was selected for further study.

Next, the impact of pMHC valency on the binding avidity of Dor-pMHCs was investigated. We fabricated another two series of Dor-pMHCs with a fixed inter-pMHC spacing of 20 nm, including 1D Dor-*nx*-20 bearing 1–5 of SA-binding sites that are distributed equidistantly on one edge (Fig. 2a, middle) and 2D Dor-*nx*-20 bearing 3–18 of SA-binding sites that are distributed equidistantly on three edges (Fig. 2a, right). The successful assembly of these two series of Dor-pMHCs was verified by AFM imaging (Fig. 2b and Supplementary Fig. 10). With increasing the pMHC valency, Dor-pMHCs exhibited higher binding avidity with apparent *K*_D,N_ decreasing from 86.9 to 10.5 nM for 1D Dor-*nx*-20 (Fig. 2b and Supplementary Fig. 11b) and decreasing from 20.7 to 1.8 nM for 2D Dor-*nx*-20 (Fig. 2b and Supplementary Fig. 11c), which are comparable to or lower than those of common pMHC multimers, including tetramer (*K*_D,N_ = 60.7 nM) or dextramer (*K*_D,N_ = 18.2 nM) (Supplementary Fig. 11d, e).

To gain deep insight into the function of the pMHC valency on their binding avidity, the obtained apparent *K*_D,N_ was plotted against the pMHC valency of Dor-pMHCs (Supplementary Fig. 12). Here, the cooperativity parameter (*α*) was used to quantify the activity of the multivalency effect by Eq. (1)^40, 41^

1 $${K}_{{{{{{\rm{D}}}}}},{{{{{\rm{N}}}}}}}={({K}_{{{{{{\rm{D}}}}}},1})}^{{{{{{\rm{\alpha }}}}}}N}$$

where apparent *K*_D,N_ is the Dor-pMHCs’ binding avidity, *K*_D,1_ is the pMHC monomer’s binding affinity, and *N* is the pMHC valency. Multivalent interactions are identified as positively cooperative when *α* > 1, noncooperative when *α* = 1, and negatively cooperative when *α* < 1^42^. Similar to other well-characterized multivalent scaffolds^42, 43^, the Dor-pMHCs exhibited values of *α* < 1, indicating their negative cooperativity (Supplementary Table 1), which possibly arises from steric hindrance. The contribution of the multivalent interaction in relation to the monovalent interaction can also be quantified by the enhancement parameter (*β*) using Eq. (2)^43^

2 $${K}_{{{{{{\rm{D}}}}}},{{{{{\rm{N}}}}}}}={{\beta }}{K}_{{{{{{\rm{D}}}}}},1}$$

The values of 1/*β* indicated that 2D Dor-*nx*-20 is more sensitive to the increase of pMHC valency than the other two series (Supplementary Table 1), suggesting that the contribution of each individual binding epitope is significant in 2D Dor-*nx*-20^44^. Such multivalent display of pMHC molecules in 2D pMHC multimers facilitates their robust binding to T cells.

We next measured the apparent dissociation rates of 2D Dor-*nx*-20 to evaluate their binding stability. For OT-1 naive T cells, the tetramers bound TCRs with a half-life (*t*_1/2_) of 34.7 min and apparent dissociation rate (*k*_off_) of 3.33 × 10^−4^ s^−1^, whereas the dextramers bound TCRs with a *t*_1/2_ of 56.7 min and *k*_off_ of 2.05 × 10^−4 ^s^−1^ (Fig. 2c and Supplementary Fig. 13). Compared with tetramers and dextramers, the 2D pMHC multimers exhibited much slower dissociation rates decreasing from 2.55 × 10^−4^ to 0.56 × 10^−4^ s^−1^ (Supplementary Fig. 13 and Supplementary Table 2), and their corresponding *t*_1/2_ increased from 45.3 to 208.2 min (Fig. 2c). Moreover, their binding stability was found to be positively correlated to the pMHC valency. For example, 2D Dor-18*x*-20 with the highest pMHC stoichiometry exhibited the lowest *k*_off_ of 0.56 × 10^−4^ s^−1^, which equates to 6.0-fold and 3.6-fold slower *k*_off_ of the tetramer and the dextramer, respectively. These results reflect that 2D pMHC multimers with relatively short inter-pMHC distance (20 nm) and high pMHC stoichiometry (9–54 copies) show high-binding avidity and robust-binding stability to T cells, laying the foundation for detecting rare and low-affinity antigen-specific T cells. We refer to such 2D pMHC multimers (i.e., 2D Dor-*nx*-20) as dorimers in the following applications.

### Detection of OVA-specific CD8^+^ T cells

**Fig. 3:**
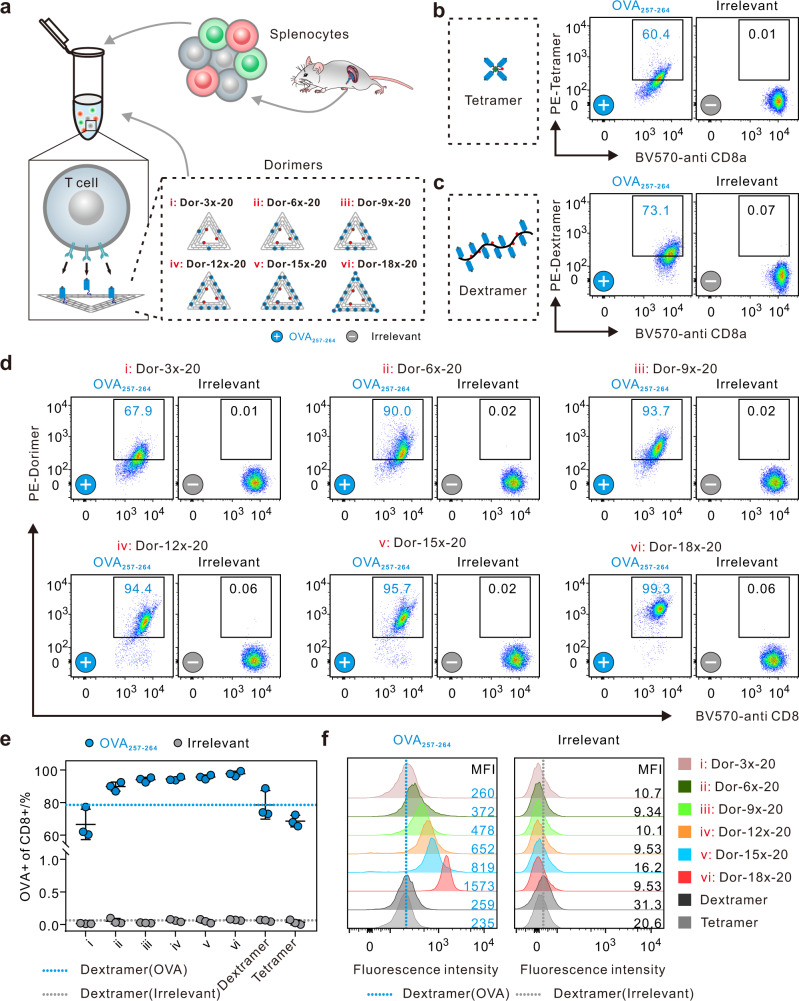
Detection of OVA-specific CD8^+^ T cells using dorimers. **a** Schematic illustrating phenotype analysis of OVA-specific CD8^**+**^ T cells from OT-1 transgenic mice with six types of dorimers (i–vi). Antigen-specific staining of OVA-specific CD8^**+**^ T cells by 3 nM pMHC multimers, including **b** dormers, **c** tetramers and **d** dextramers. pMHC multimers were made using H-2K^b^ MHC loaded with the specific OVA peptide (SIINFEKL) (**+**) or an irrelevant peptide (SIYRYYGL) (**−**). The number in each panel represents the percentage of the identified OVA-specific CD8^**+**^ T cells. **e** Statistical analysis of the percentages of the identified OVA-specific CD8^**+**^ T cells by tetramers, dextramer, and dorimers at 3 nM. The blue and gray lines represent the percentage of OVA-specific CD8^**+**^ T cells detected by the OVA_257–264_/irrelevant-dextramer, respectively. Data represent the mean ± s.d. from *n* = 3 independent experiments. Source data are provided as a Source Data file. **f** The mean fluorescence intensity (MFI) of OVA-specific CD8^**+**^ T cells after incubation with PE-conjugated tetramers, dextramer, and dorimers at 3 nM. Left: OVA peptide (SIINFEKL); Right: irrelevant peptide (SIYRYYGL).

We firstly utilized the dorimers (i.e., Dor-*nx*-20) made using H-2K^b^ MHC loaded with an OVA_257–264_ peptide (SIINFEKL) for detection of high-frequency antigen-specific T cells, such as ovalbumin (OVA)-specific CD8^**+**^ T cells extracted from the spleen of OT-1 transgenic mice (Fig. 3a, Supplementary Fig. 14). Compared with tetramers and dextramers (identified 60.4% and 73.1% OVA-specific CD8^**+**^ T cells, Fig. 3b, c)^45–48^, dorimers could significantly augment the staining efficiency for identification of high-frequency antigen-specific T cells at the low concentration (3 nM, Fig. 3d, e, Supplementary Table 3). Over 90% OVA-specific CD8^**+**^ T cells were identified from the total CD8^**+**^ T cells using Dor-*nx*-20, and up to 99.3% were detected with increasing the pMHC valency, except that only ∼68% was detected by Dor-3*x*-20. On the contrary, almost no nonspecific staining (≤0.06%) could be identified from the CD8^**+**^ T cells in the negative control group (H-2K^b^, irrelevant peptide, SIYRYYGL), implying the low background of dorimers in detecting antigen-specific T cells. These results were also supported by the mean fluorescence intensity of pMHC multimers staining (Fig. 3f). Moreover, this staining was specific to OVA-specific CD8^**+**^ T cells and negligible nonspecific bindings to CD4^**+**^ T cells were observed (Supplementary Fig. 15). Also, only a very few OVA-specific CD8^**+**^ T cells extracted from non-transgenic C57BL/6 mice were identified by these pMHC multimers (Supplementary Fig. 16). Furthermore, when using more tetramers (up to 60 nM) or dextramers (up to 30 nM) for staining OVA-specific CD8^+^ T cells from OT-1 transgenic mice, despite of the improved detection limit, the nonspecific bind would also be increased (Supplementary Fig. 17, Supplementary Table 4). Additionally, dorimers are significantly better than tetramers and dextramers with equivalent pMHC concentration in both staining efficiency and background (detailed analysis in Supplementary Table 5). Furthermore, we confirmed that dorimers can be applied for analysis of human cytomegalovirus (CMV)-specific CD8^+^ T cells from peripheral blood mononuclear cells (PBMCs) (see Supporting Information for more detailed analysis, Supplementary Figs. 18–21).

### Detection of double-positive CD4^+^CD8^+^ T cells

**Fig. 4:**
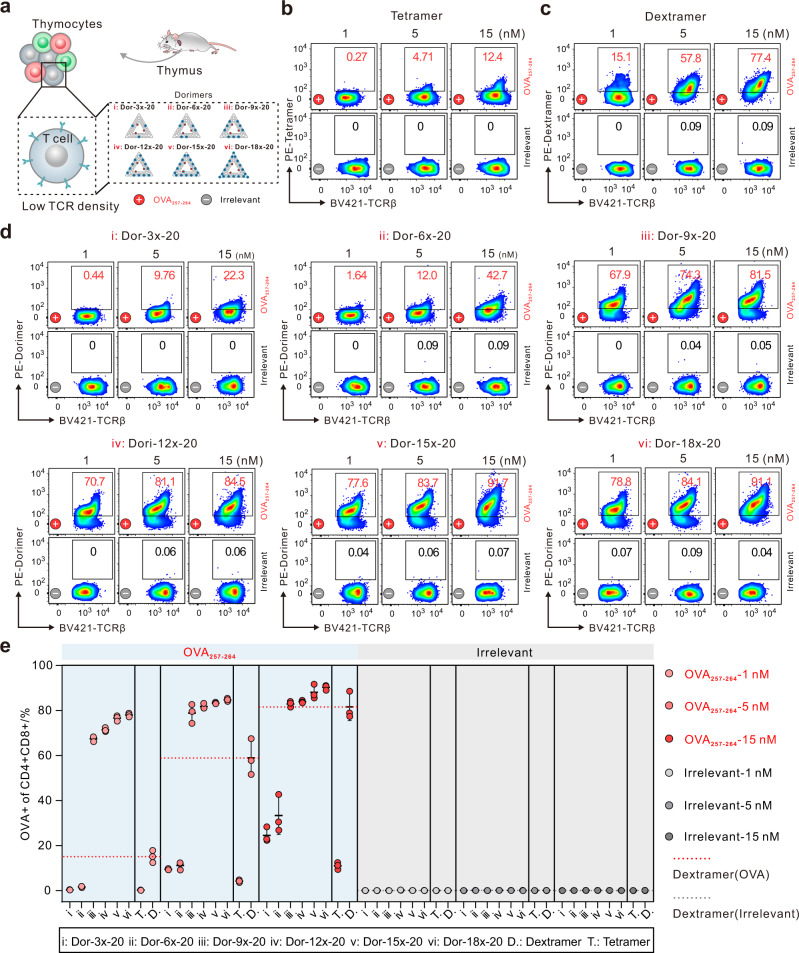
Detection of double-positive CD4^+^CD8^+^ T cells using dorimers. **a** Schematic illustrating phenotype analysis of OVA-specific CD4^**+**^CD8^**+**^ T cells from OT-1 transgenic mice thymus with six types of dorimers (i–vi). Antigen-specific staining of OVA-specific CD4^**+**^CD8^**+**^ T cells by **b** tetramers, **c** dextramer, and **d** dorimers at different concentrations (1, 5, and 15 nM). pMHC multimers were made using H-2K^b^ MHC loaded with either a specific OVA peptide (SIINFEKL) (**+**) or an irrelevant peptide (SIYRYYGL) (**−**). The number in each panel represents the percentage of the identified OVA-specific CD8^**+**^ T cells. **e** Statistical analysis of the percentages of the identified OVA-specific CD4^**+**^CD8^**+**^ double-positive T cells from OT-1 transgenic mice by dorimers, tetramers, and dextramer at different concentrations (1, 5, and 15 nM). The red and gray lines represent the percentage of OVA-specific CD8^**+**^ T cells detected by the OVA_257–264_/irrelevant-dextramer, respectively. Blue shading: OVA peptide (SIINFEKL); Gray shading: irrelevant peptide (SIYRYYGL). Data represent the mean ± s.d. from *n* = 3 independent experiments. Source data are provided as a Source Data file.

Dorimers were then employed for staining populations of low avidity T cells (Fig. 4a). Here, the immature double-positive CD4^**+**^CD8^**+**^ T thymocytes on which TCRs are 10- to 30-fold less dense than mature T cells were used as the study model^49^. After antigen-specific staining of CD4^**+**^CD8^**+**^ T cells extracted from OT-1 transgenic mice (Supplementary Fig. 22), only 12.4% OVA-specific CD4^**+**^CD8^**+**^ T cells could be stained even using 15 nM OVA/H-2K^b^ tetramers (Fig. 4b), whereas 57.8% were identified by 5 nM OVA/H-2K^b^ dextramers and 77.4% were identified by 15 nM OVA/H-2K^b^ dextramers (Fig. 4c). Notably, we found that increasing the pMHC valency of dorimers led to a monotonic increase in the detection efficiency (Fig. 4d, e). For instance, 78.8% OVA-specific CD4^**+**^CD8^**+**^ T cells were identified by Dor-18*x*-20 at 1 nM, which is even better than the OVA/H-2K^b^ dextramers at 15 nM (77.4%). More importantly, by further increasing the concentration up to 15 nM, the frequency of OVA-specific CD4^**+**^CD8^**+**^ T cells could be improved to 91.1% by Dor-18*x*-20 (Fig. 4d, e), which is superior than equivalent tetramers and dextramers (Supplementary Table 6). These results were also confirmed by the staining intensities of OVA-specific CD4^**+**^CD8^**+**^ T cells (Supplementary Fig. 23). Additionally, dorimers showed higher frequency of OVA-specific CD4^**+**^CD8^**+**^ T cells and less nonspecific binding in comparison to tetramers and dextramers with equivalent pMHC concentration (detailed analysis in Supplementary Table 8, Supplementary Fig. 24), which is consistent with the results for OVA-specific CD8^**+**^ T cells. Furthermore, a small number of OVA-specific CD4^**+**^CD8^**+**^ thymocytes (<1.6%) extracted from non-transgenic C57BL/6 mice could be detected by these pMHC multimers (Supplementary Fig. 25).

### Detection of autoimmune CD8^+^ T cells

**Fig. 5:**
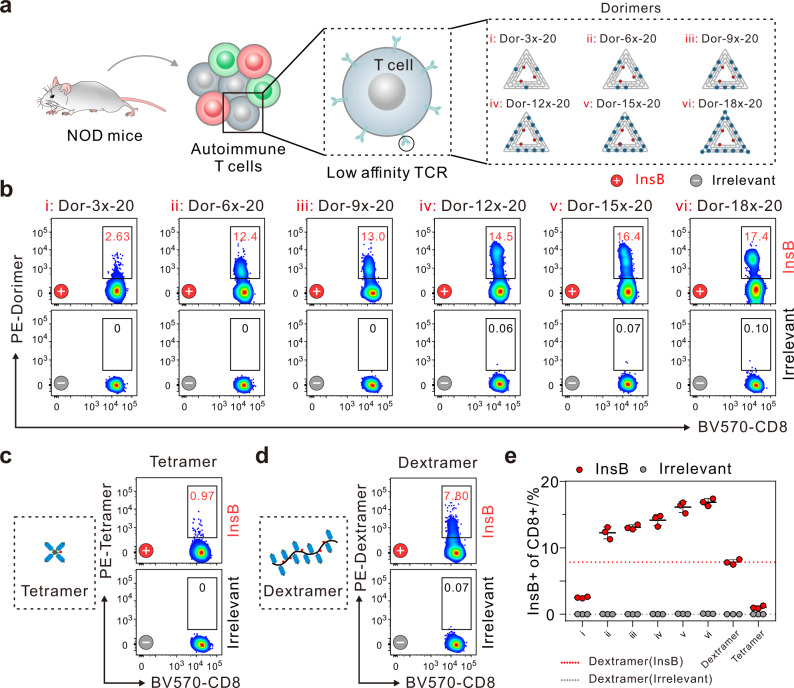
Detection of autoimmune CD8^+^ T cells using dorimers. **a** Schematic illustrating phenotype analysis of InsB-specific CD8^**+**^ T cells with six types of dorimers (i–vi). Antigen-specific staining of InsB-specific CD8^**+**^ T cells derived from the mice spleen at 8 weeks by **b** dorimers, **c** tetramer, and **d** dextramer at 10 nM. pMHC multimers were made using H-2K^d^ MHC loaded with either a specific InsB peptide (LYLVCGERL) (**+**) or an irrelevant TUM peptide (KYQAVTTTL) (**−**). The number in each panel represents the percentage of the identified InsB-specific CD8^**+**^ T cells. **e** Statistical analysis of the percentages of the identified InsB-positive CD8^**+**^ T cells by dorimers, tetramer and dextramer at 10 nM. The red and gray lines represent the percentage of InsB-specific CD8^**+**^ T cells detected by the InsB-/irrelevant-dextramer, respectively. Data represent the mean ± s.d. from *n* = 3 independent experiments. Source data are provided as a Source Data file.

Dorimers were next applied for identification of autoimmune CD8^**+**^ T cells from the spleen of nonobese diabetic (NOD) mice that is an extensively study model of human type 1 diabetes (Fig. 5a)^50–52^. Due to the low-affinity TCRs, autoimmune T cells are elusive to be directly detected in peripheral blood^53, 54^. The autoimmune CD8^**+**^ T cells from NOD mice were stained by pMHC multimers made using H-2K^d^ MHC loaded with either a specific InsB peptide (LYLVCGERL) or an irrelevant TUM peptide (KYQAVTTTL) (Supplementary Fig. 26). We found that up to 17.4% InsB-specific CD8^+^ T cells could be detected by dorimers at 10 nM with increasing the pMHC valency (Fig. 3d). In contrast, 0.97% and 7.80% InsB-specific autoimmune CD8^**+**^ T cells were detected by tetramers and dextramers at 10 nM (Fig. 5c, e). Moreover, even using 60 nM tetramers, only ∼2.65% autoimmune CD8^**+**^ T cells were identified, and 11.3% autoimmune CD8^**+**^ T cells were stained by 40 nM dextramers, with no or little improvement in detection performance yet showing increased nonspecific binding (Supplementary Fig. 27). Similarly, dorimers excel in both staining efficiency and background than dextramers with equivalent pMHC concentration (detailed analysis in Supplementary Table 11).

## Discussion

In summary, we have developed DNA origami-based pMHC multimers (dorimers) for antigen-specific CD8^+^ T cell detection. The developed dorimers show several distinct advantages over other pMHC multimers. First, given the high programmability and precise addressability of nucleic acids, pMHC molecules can be precisely organized on the DNA origami, allowing for precise control over the pMHC valency (ranging from 3 to 54) and the inter-pMHC spacing (20–80 nm). Second, the binding avidity and binding stability of Dor-pMHCs to antigen-specific T cells can be tuned by altering the pMHC valency or the inter-pMHC spacing, resulting in apparent dissociation constants ranging from 1.8 to 86.9 nM and dissociation rates ranging from 2.55 × 10^−4^ to 0.56 × 10^−4^ s^−1^. Third, dorimers with high valency of pMHC present distinctive features, including high-binding avidity, slow dissociation kinetics, staining efficiency and low background, which could facilitate the detection of low avidity and low-affinity antigen-specific T cells. Fourth, dorimers can also be applied for analysis of human T cells from PBMCs, thus could find promising applications in disease management. Given these advantages, dorimers might constitute the reagents of choice for investigating antigen-specific T cells in basic research and clinical applications.

## Methods

### Mice

All animal studies were carried out in accordance with the guidelines for the care and use of laboratory animals approved by the Animal Ethics Committee of East China Normal University. C57BL/6 mice and OT-1 mice were provided by Shanghai Model Organisms Biological Technology Co., Ltd. NOD mice were provided by GemPharmmatech Biotechnology Co., Ltd. Male and female mice were used between 4 and 8 weeks of age. C57BL/6 mice and OT-1 mice were on a pure C57BL/6 genetic background. NOD mice were on a pure NOD/ShiLtJ genetic background. Mice were housed with a 12 h light/dark cycle at 22–25 °C (50–60% relative humidity), and with food and water ad libitum.

### Materials and reagents

M13mp18 DNA was purchased from New England Biolabs, Inc. (Ipswich, MA, USA). Oligonucleotides were synthesized by Sangon Biotech Co., Ltd. (Shanghai, China) with standard desalting and purified with HPLC, and used without further purification. Information on the DNA oligonucleotide sequences are provided in Supplementary Data 1. Streptavidin (SA) and PE-SA were obtained from Solarbio Science & Technology Co., Ltd. (Beijing, China). Biotin-labeled peptide–MHC monomers (H-2K^b^-OVA_257-264_, H-2K^d^-InsB, HLA-A*0201 CMV pp65, etc.) and tetramers were provided by MBL Biotech Co., Ltd (Japan). Dextramer was provided by Immudex (Copenhagen, Denmark). Fluorescent anti-mouse/human antibodies used for flow cytometry analysis were purchased from BioLegend. MTT Cell Proliferation and Cytotoxicity Assay Kit (cat. 606334) were provided by Sangon Biotech Co., Ltd. (Shanghai, China). All other chemicals were obtained from Sinopharm and Sigma-Aldrich.

### Assembly of biotinylated DNA origami

Typically, the procedure for assembly of biotinylated DNA origami was as follows: M13mp18 and short DNA staple strands (including biotinylated DNA and overhangs DNA) at a molar ratio of 1:10 were mixed in 1×TAE–Mg^2+^ buffer (40 mM Tris, 20 mM acetic acid, 2 mM EDTA, and 12.5 mM magnesium acetate, pH = 8.0). Biotinylated DNA origami was assembled by heating to 95 °C for 5 min and slowly cooling down from 95 to 25 °C for 7000 s using a PTC-200 Peltier Thermal Cycler. Biotinylated DNA origami was subsequently purified with Millipore Amicon Ultra 100 kDa spin columns to remove excess staple strands, and this process was repeated three times. The purified biotinylated DNA origami was quantified by determining its absorption at 260 nm and stored at 4 °C for further use.

### Fabrication of Dor-pMHCs

Prior to fabrication of Dor-pMHCs, Dor-SA was assembled by incubating biotinylated DNA origami with SA for 30 min at a molar ratio of 1:5*n* (*n* indicates the number of SA-binding site). The assembled Dor-SA was then dispersed into 1×TAE buffer after removing excess SA by ultrafiltration, and mixed with biotinylated pMHC molecules at a molar ratio of 1:10*n* and incubated for 30 min. After removing excess biotinylated pMHC molecules by ultrafiltration, the Dor-pMHCs were dispersed in 1×TAE buffer, quantified by determining its absorption at 260 nm, and stored at 4 °C for further use.

### Fabrication of PE-labeled Dor-pMHCs

Prior to fabrication of PE-labeled Dor-pMHCs, fluorescent protein phycoerythrin (PE)-labeled poly(A) DNA was prepared by mixing 5’biotin-poly(A) DNA with PE-labeled streptavidin at a molar ratio of 4:1 and incubating for 30 min. Then, Dor-pMHCs with overhang strands (poly(T) DNA) were incubated with PE-labeled poly(A) DNA for 30 min at a mole ratio of 1:10*n* (*n* indicates the stoichiometric number of overhangs). Removing excess PE-labeled poly(A) DNA by ultrafiltration yielded PE-labeled Dor-pMHCs. The fluorescence intensity of PE-labeled Dor-pMHCs was measured with flow cytometry.

### Agarose electrophoresis

The 1% agarose gels were prepared with 1×TAE-Mg^2+^ buffer (40 mM Tris, 20 mM acetic acid, 2 mM EDTA, and 12.5 mM MgCl_2_, pH = 8.0), run at 90 V for 90 min, and visualized under UV light. Finally, the band was visualized on a gel imaging system (Tanon Science & Technology Co., Ltd., China).

### AFM imaging

The assembled Dor-pMHCs (or biotinylated DNA origami, Dor-SA) were characterized with AFM (a Multimode atomic force microscope, Bruker, USA). For AFM imaging, 5 μL of diluted sample solution was deposited on the fresh mica for 3 min, and the substrate was then washed with distilled water to remove the unabsorbed samples, followed by drying with high-purity nitrogen. The prepared samples were scanned with ScanAsyst-Air tips at 0.4 N/m spring constant under ScanAsyst Mode and a resolution of 512 pixels per line with 2 Hz scan rate.

### Stability study of Dor-pMHCs

To study their structural stability, Dor-pMHCs were incubated in 1×PBS at 25 or 37 °C. At a preset time (0, 4, 8, 12, 24, 48, 72 h), AGE was carried out for analyzing the structural integrity of Dor-pMHCs.

### Cell extraction

Mouse splenocytes and thymocytes were harvested from C57BL/6 (or OT-1 mice, NOD mice) and cultured in RPMI 1640 medium containing 10% FBS and 1% penicillin–streptomycin at 37 °C in a humidified atmosphere with 5% CO_2_. The frozen human PBMCs (stored in liquid nitrogen before use) from 10 HLA-A2 positive healthy donors that were nonidentifiable were purchased from Miao Tong Biotechnology Co., Ltd. (Shanghai, China). All donors provided written informed consent permitting PBMCs collection and correlative studies. These samples were serotyped for identification of CMV reactivation/infection status. All experiments involving cells were conducted at 4 °C unless otherwise stated.

### Cytotoxicity evaluation

The splenocytes extracted from C57BL/6 mice were seeded in a 96-well plate at a density of 1 × 10^4^ per well. After culturing overnight, the extracted cells were exposed to different concentrations of Dor-pMHCs (1, 5, 10, and 20 nM) for 12 h, followed by adding 20 μL of the MTT (5 mg mL^−1^) into each well for 4 h incubation. After removing the medium, the cells were mixed with 150 μL of dimethyl sulfoxide and their absorbance was recorded at a test wavelength of 570 nm and a reference wavelength of 630 nm by a microplate reader (Varioskan LUX, USA).

### Determination of apparent dissociation constant (*K*_D,N_) and dissociation rate (*k*_off_)

To quantify the binding avidity of Dor-pMHCs (or tetramer, dextramer) to OVA-specific CD8^**+**^ T cells, various concentrations of PE-labeled Dor-pMHCs from 0 to 120 nM (each Dor-pMHCs labeled with three PE molecules) were incubated with 1 × 10^6^ cells/mL for 30 min in the presence of BV570-labeled anti-CD8 (Biolegend, cat #: 100739, clone 53-6.7, 1:100 dilution), FITC-labeled anti-CD3 (Biolegend, cat. #100204, clone 17A2, 1:100 dilution), and aqua live/dead cell stain. Then, the free PE-labeled 2D Dor-*nx*-20 were removed by centrifugation. Following, the fluorescence intensity of the cell population was measured with flow cytometry (Merck Millipore flowSight cytometer, Germany), and the fluorescence intensity versus concentration was plotted to generate a saturation binding curve for each sample. Finally, apparent dissociation constant (*K*_D,N_) was calculated by fitting binding curve with Origin 8.0 Pro.

For determining dissociation rate (*k*_off_), OT-1 splenocytes were incubated with 3 nM PE-labeled 2D Dor-*nx*-20 (or tetramer, dextramer) in the presence of BV570-labeled anti-CD8 (Biolegend, cat. #100739, clone 53-6.7, 1:100 dilution), FITC-labeled anti-CD3 (Biolegend, cat. #100204, clone 17A2, 1:100 dilution), and aqua live/dead cell stain at 4 °C for 1 h. After centrifugation, cells were pelleted and resuspended in 100 μL FACS buffer in the presence of 50 μg/mL anti-H-2K^b^ MHC antibody (Biolegend, cat#: 114602, clone 28-8-6). At preset time points, the fluorescence of cells was measured with flow cytometry after adding the anti-H-2K^b^ MHC-blocking antibody. Finally, the data were analyzed with FlowJo software, and a first-order decay kinetic model was used to fit the plot, yielding *k*_off_ and *t*_1/2_.

### Detection of antigen-specific T cells

For determining antigen-specific T cells, the splenocytes extracted from C57BL/6 mice (OT-1 mice) at 6–8 weeks were firstly incubated in flowing cytometry staining buffer (1×PBS, 2% FCS, 0.1% NaN_3_) with 1% anti-CD16/CD32 Fc blocker (Biolegend, cat. #101301, clone 93, 1:100 dilution) at 4 °C for 30 min, and then stained with PE-labeled dorimers or tetramers (dextramers) and an antibody mixture containing BV570-labeled anti-CD8 (Biolegend, cat. #100739, clone 53-6.7, 1:100 dilution), PerCP/Cyanine5.5-labeled anti-CD4 (Biolegend, cat. #100434, clone GK1.5, 1:100 dilution), FITC-labeled anti-CD3 (Biolegend, cat. #100204, clone 17A2, 1:100 dilution), anti-F4/80 (Biolegend, cat. #123101, clone BM8, 1:100 dilution), anti-Ly-6G/Ly-6C (Gr1) (Biolegend, cat. #108401, clone RB6-8C5, 1:100 dilution), anti-CD11c (Biolegend, cat. #117301, clone N418, 1:100 dilution), anti-CD11b (Biolegend, cat. #101201, clone M1/70, 1:100 dilution), and aqua live/dead cell stain at 4 °C for 30 min. For determining CD4^**+**^CD8^**+**^ thymocytes, the thymocytes extracted from C57BL/6 mice (or OT-1 mice) at 4–5 weeks were stained with PE-labeled dorimers (tetramers or dextramers) and an antibody mixture containing BV570-labeled anti-CD8 (Biolegend, cat. #100739, clone 53-6.7, 1:100 dilution), PerCP/Cyanine5.5-labeled anti-CD4 (Biolegend, cat. #100434, clone GK1.5, 1:100 dilution), FITC-labeled anti-CD3 (Biolegend, cat. #100204, clone 17A2, 1:100 dilution), BV421-labeled anti-TCRβ (Biolegend, cat. #109229, clone H57-597, 1:100 dilution), and aqua live/dead cell stain at 4 °C for 30 min, in which dorimers, tetramer and dextramer were prepared using H-2K^b^ MHC loaded with either an OVA peptide (SIINFEKL) or an irrelevant peptide (SIYRYYGL). For staining autoimmune T cells from a type I diabetic NOD mice using dorimers or tetramers. The splenocytes extracted from the NOD mice at 8 weeks were incubated with dorimers (or tetramers) and co-stained with an antibody mixture including BV570-labeled anti-CD8 (Biolegend, cat. #100739, clone 53-6.7, 1:100 dilution), PerCP/Cyanine5.5-labeled anti-CD4 (Biolegend, cat. #100434, clone GK1.5, 1:100 dilution), FITC-labeled anti-CD3 (Biolegend, cat. #100204, clone 17A2, 1:100 dilution) and aqua live/dead cell stain at 4 °C for 30 min, in which dorimers and tetramers were prepared using H-2K^d^ MHC loaded with either an InsB peptide (LYLVCGERL) or an irrelevant TUM peptide (KYQAVTTTL). Frozen human PBMCs were thawed in a water bath at 37 °C and rested in RPMI 1640 medium containing 10% FBS and 1% penicillin–streptomycin for 1 h at 37 °C. For staining human CD8^**+**^ T cells, human PBMCs were incubated with dorimers (or tetramers) and co-stained with an antibody mixture including Pacific Blue-labeled anti-CD8 (Biolegend, cat. #344717, clone SK1, 1:100 dilution), FITC-labeled anti-CD3 (Biolegend, cat. #317306, clone OKT3, 1:100 dilution), PerCP/Cy5.5-labeled anti-CD4 (Biolegend, cat. #317427, clone OKT4, 1:100 dilution), anti-CD19 (Biolegend, cat. #302202, clone HIB19, 1:100 dilution), and aqua live/dead cell stain. HLA-A2 molecules loaded with human cytomegalovirus (CMV) peptides (NLVPMVATV) or irrelevant HIV peptides (SLYNTVATL) were used for the staining of human antigen-specific CD8^**+**^ T cells. After staining for 30 min at room temperature in the dark, the cells were repeatedly washed and resuspended in 100 μL of FACS buffer. Finally, the harvested samples were analyzed with flow cytometry.

### Statistics and reproducibility

All AFM images presented in the manuscript and the Supplementary Information are exemplary micrographs and show representative images of many acquired micrographs. All agarose gel electrophoresis experiments were repeated independently multiple times and reliably reproduced the same results. The number of replicates for other experiments is provided in the caption of each figure.

### Reporting summary

Further information on research design is available in the Nature Research Reporting Summary linked to this article.

## Supplementary information

Supplementary Information

Description of Additional Supplementary Files

Supplementary Dataset 1

Reporting Summary

## Data Availability

All other data are available in the article and its Supplementary files or from the corresponding author upon reasonable request. Source data are provided with this paper.

## Source data

Source data
