# Supplementary material for: Nanoscale organization of two-dimensional multimeric pMHC reagents with DNA origami for CD8+ T cell detection
Source: Nat Commun. 2022 Jul 7;13:3916. doi: 10.1038/s41467-022-31684-8 (PMC9263106; doi:10.1038/s41467-022-31684-8)
Supplement: Supplementary file 1 — Supplementary Information [file 41467_2022_31684_MOESM1_ESM.pdf]

Supplementary Information

Nanoscale organization of two-dimensional multimeric pMHC reagents with DNA origami for CD8+ T cell detection

Yueyang Sun[1], Lu Yan[1], Jiajia Sun[1], Mingshu Xiao[1], Wei Lai[1], Guangqi Song[2], Li Li[1], Chunhai Fan[3] and Hao Pei[1,4]*

[1]Shanghai Key Laboratory of Green Chemistry and Chemical Processes, School of Chemistry and Molecular Engineering, East China Normal University, 500 Dongchuan Road, Shanghai 200241, China

[2]Department of Gastroenterology and Hepatology, Zhongshan Hospital of Fudan University, Shanghai, China

[3]School of Chemistry and Chemical Engineering, and Institute of Molecular Medicine, Renji Hospital, School of Medicine, Shanghai Jiao Tong University, Shanghai 200240, China

[4]Institute of Eco-Chongming, Shanghai 202162, China

*Corresponding author. Email: peihao@chem.ecnu.edu.cn

Table of contents

Detection of CMV-specific $CD8^+$ T cells. 1
Supplementary Fig. 1 2
Supplementary Fig. 2 3
Supplementary Fig. 3 4
Supplementary Fig. 4 5
Supplementary Fig. 5 6
Supplementary Fig. 6 7
Supplementary Fig. 7 8
Supplementary Fig. 8 9
Supplementary Fig. 9 10
Supplementary Fig. 10 11
Supplementary Fig. 11 12
Supplementary Fig. 12 13
Supplementary Fig. 13 14
Supplementary Fig. 14 15
Supplementary Fig. 15 16
Supplementary Fig. 16 17
Supplementary Fig. 17 18
Supplementary Fig. 18 19
Supplementary Fig. 19 20
Supplementary Fig. 20 21
Supplementary Fig. 21 22
Supplementary Fig. 22 23
Supplementary Fig. 23 24
Supplementary Fig. 24 25
Supplementary Fig. 25 26
Supplementary Fig. 26 27
Supplementary Fig. 27 28
Supplementary Table 1. 29
Supplementary Table 2. 30
Supplementary Table 3. 31
Supplementary Table 4. 32
Supplementary Table 5. 33
Supplementary Table 6. 34
Supplementary Table 7. 35
Supplementary Table 8. 36
Supplementary Table 9. 37
Supplementary Table 10 38
Supplementary Table 11. 39

Detection of CMV-specific CD8$^+$ T cells. One of prominent applications of pMHC multimers involves the detection of antigen-specific T cells from human blood samples. Human cytomegalovirus (CMV) reactivation/infection represents a critical viral infection in patients following transplant. Monitoring the immune reconstitution of CMV-specific T cells could facilitate to reduce the risks of CMV infection in post-transplant patients. In this work, we employed the dorimers for detection of CMV-specific CD8$^+$ T cells from peripheral blood mononuclear cells (PBMCs). Ten PBMCs samples (numbered as **1** to **10**), in which seven samples (Number **1** to Number **7**) were CMV-positive and the other three samples (Number **8** to Number **10**) were CMV-negative, were analyzed by exploiting HLA-A2: CMV dorimers for staining CMV-specific CD8$^+$ T cells (Supplementary Fig. 18). As expected, the negative samples were detected to show significantly lower percentage of CMV-specific CD8$^+$ T cells than that of the positive samples (Supplementary Figs. 19-20). Meanwhile, the control HLA-A2: HIV dorimers produced negligible nonspecific staining similar to that of the control HLA-A2: HIV tetramers (Supplementary Fig. 21). These observations suggest that dorimers hold great potential as an auxiliary tool for assessment of T cell-related diseases.

Figures and Tables

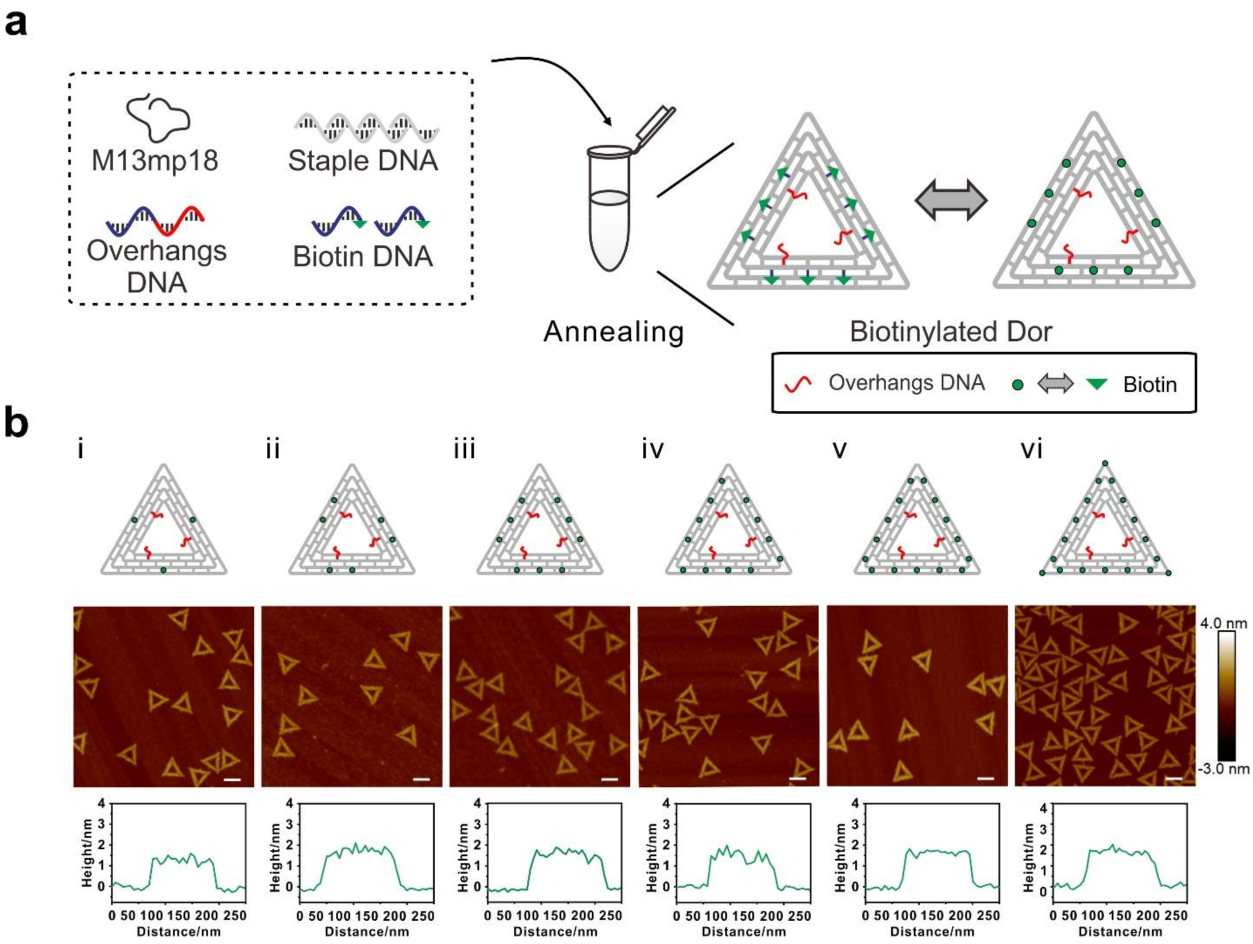

Supplementary Fig. 1. (a) Schematic illustration of fabrication of biotinylated DNA origami. **(b)** Scheme of six types of biotinylated DNA origami (i-vi: SA binding sites ranging from 3 to 18, inter-spacing of SA binding sites: 20 nm) and their corresponding AFM images and cross-section height analysis. Scale bar: 100 nm. (Dor: DNA origami)

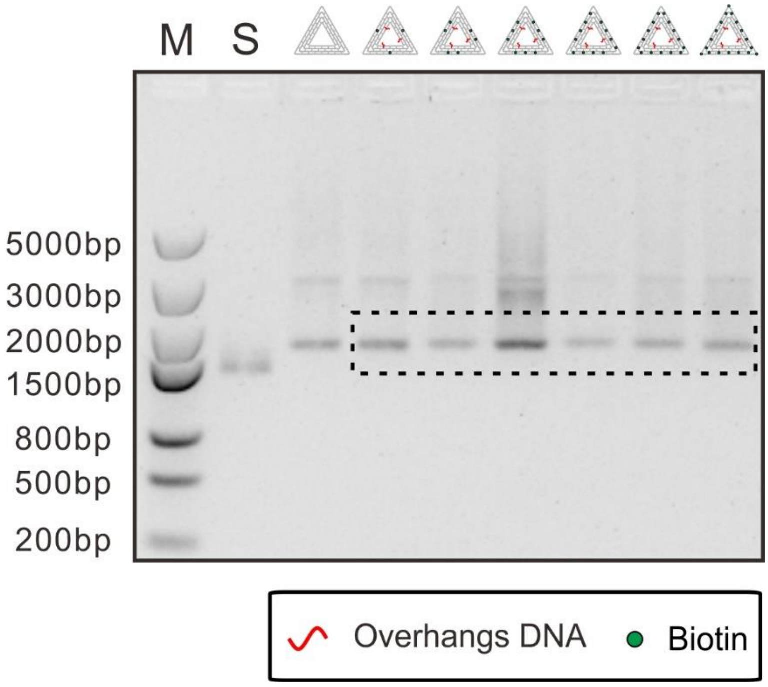

Supplementary Fig. 2. Characterization of the formation of six types of biotinylated DNA origami products by 1% agarose gel electrophoresis. The rectangle indicates the bands of the biotinylated DNA origami products. From left to right: DNA ladder (M), M13mp18 scaffold (S), triangular DNA origami, and six types of biotinylated DNA origami. Source data are provided as a Source Data file.

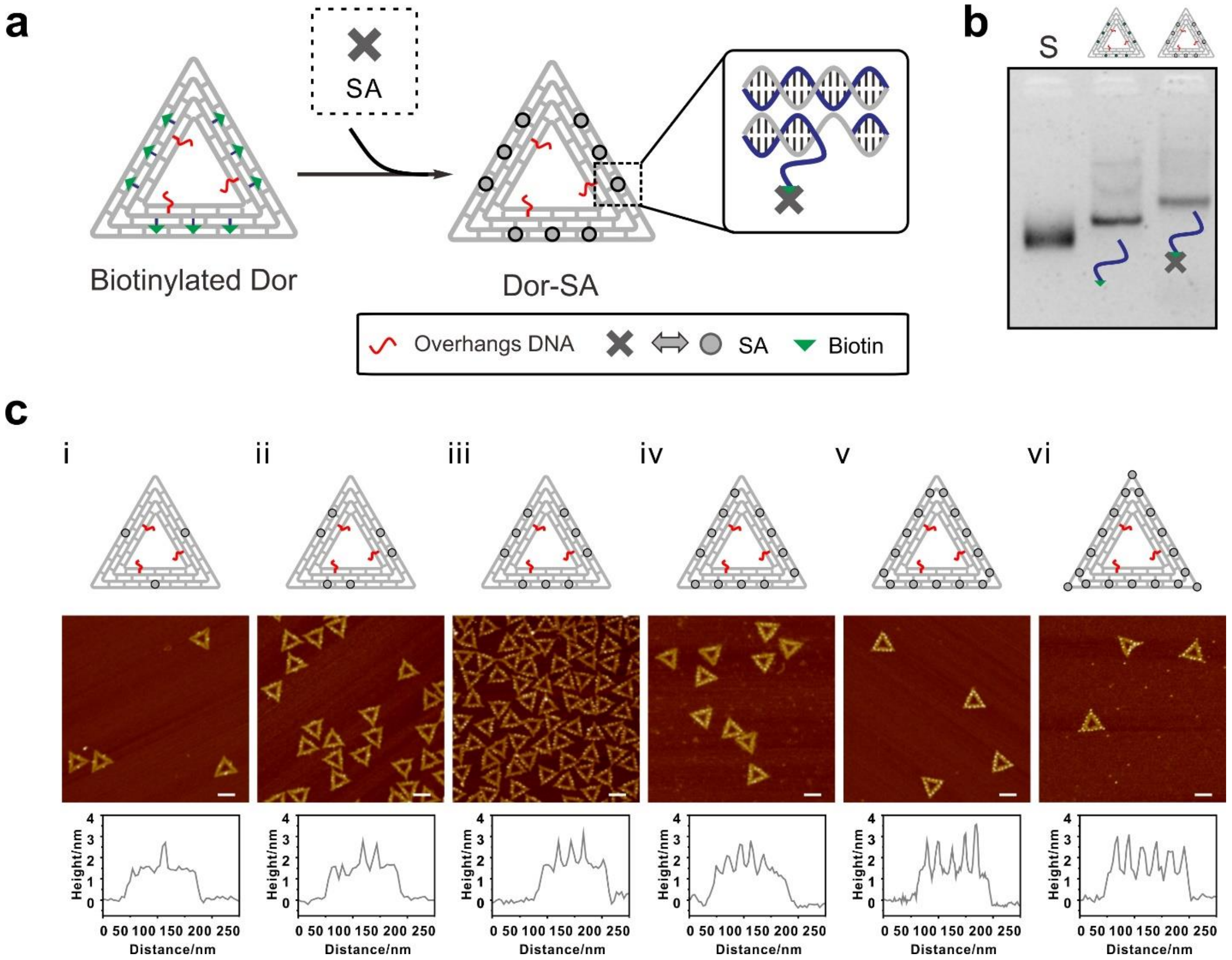

Supplementary Fig. 3. (a) Schematic illustration of SA molecules binding to biotinylated DNA origami. **(b)** Characterization of the formation of Dor-SA by 1% agarose gel electrophoresis. From left to right: M13mp18 (S), biotinylated DNA origami, Dor-SA. Source data are provided as a Source Data file. **(c)** Scheme of six types of Dor-SA (i-vi: SA number ranging from 3 to 18, inter-spacing of SA: 20 nm) and their corresponding AFM images and cross-section height analysis. Scale bar: 100 nm. (Dor: DNA origami; SA: Streptavidin)

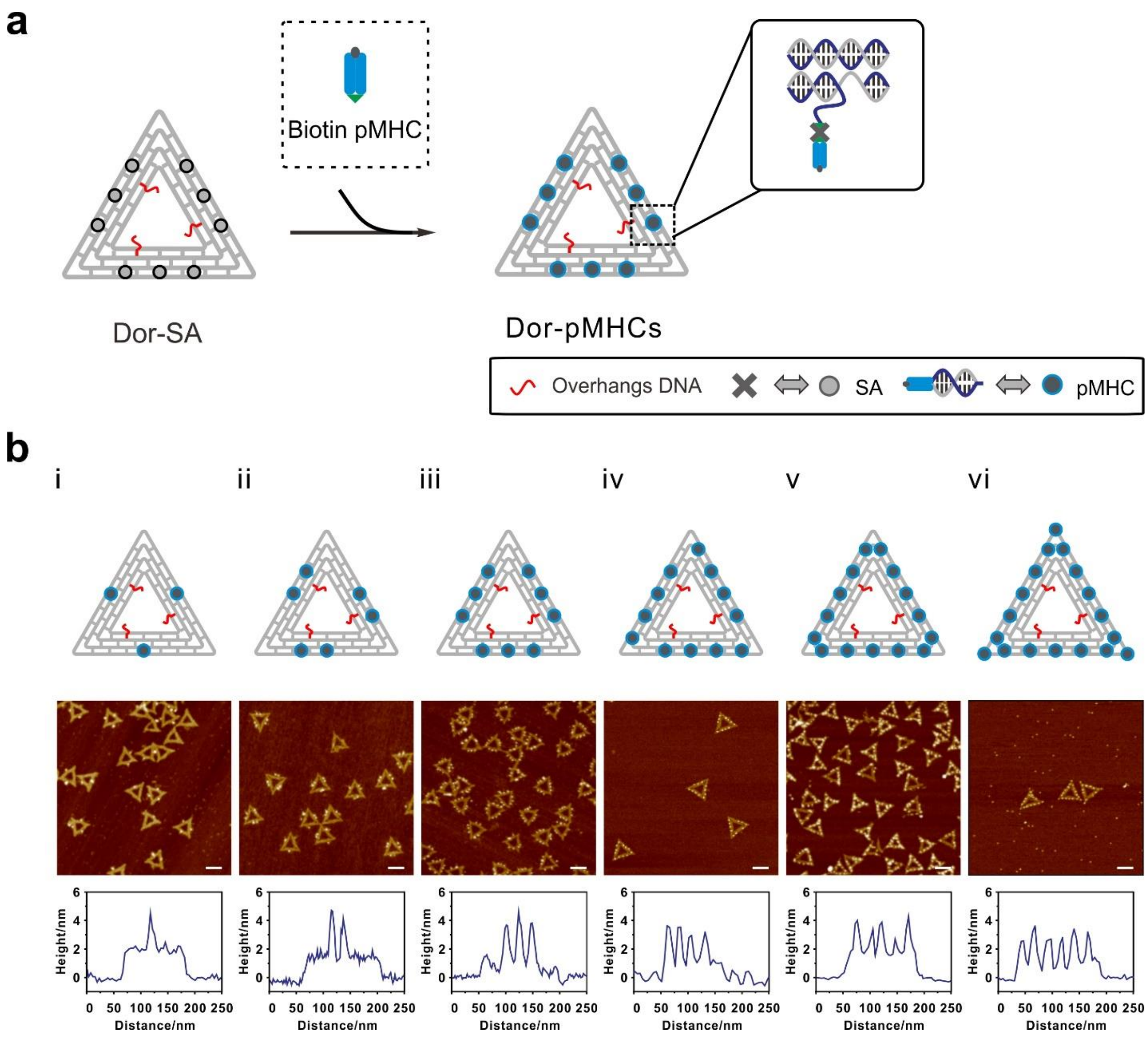

Supplementary Fig. 4. (a) Schematic illustration of the biotinylated pMHC molecules binding to Dor-SA. **(b)** Scheme of six types of Dor-pMHCs (i-vi: pMHC copies ranging from 9 to 54, inter-spacing of pMHC molecules: 20 nm) and their corresponding AFM images and cross-section height analysis. Scale bar: 100 nm. (Dor: DNA origami; SA: Streptavidin; pMHC: peptide-major histocompatibility complexes)

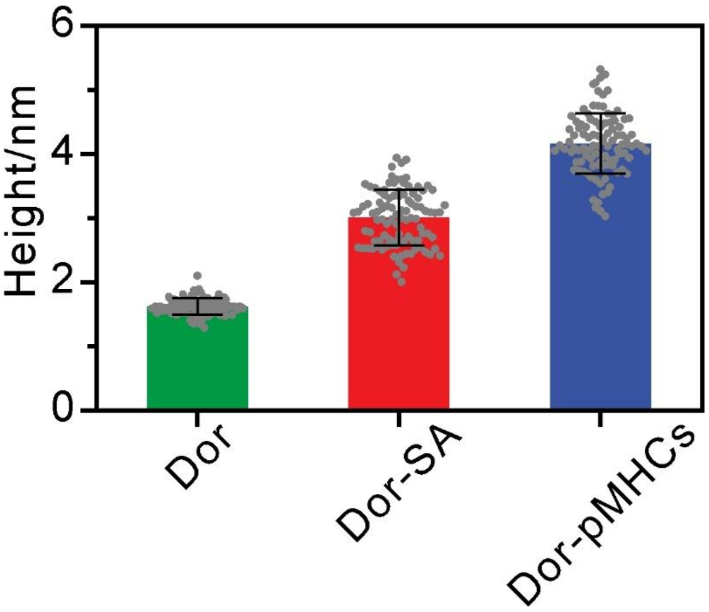

Supplementary Fig. 5. Cross-section height analysis of biotinylated DNA origami, Dor-SA and Dor-pMHCs. Data were collected based on 100 counts of samples for each group and presented as the mean ± s.d. Source data are provided as a source data file. (Dor: DNA origami; SA: Streptavidin; pMHC: peptide-major histocompatibility complexes)

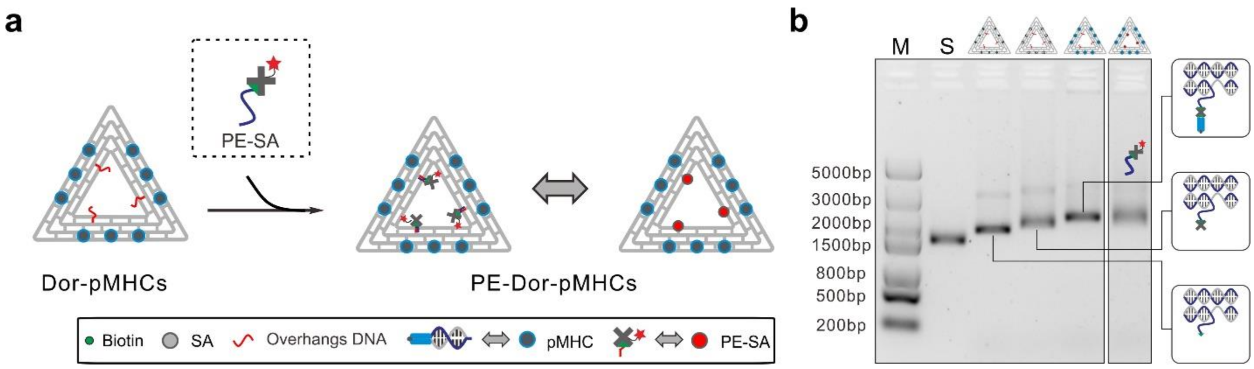

Supplementary Fig. 6. (a) Schematic illustration of the PE-SA molecules binding to Dor-pMHCs (PE-Dor-pMHCs). **(b)** Characterization of formation of PE-Dor-pMHCs by 1% agarose gel electrophoresis. From left to right: DNA ladder (M), M13mp18 scaffold (S), biotinylated DNA origami, Dor-SA, Dor-pMHCs, PE-Dor-pMHCs. Source data are provided as a Source Data file. (Dor: DNA origami; SA: Streptavidin; pMHC: peptide-major histocompatibility complexes; PE-SA: Phycoerythrin-labelled streptavidin)

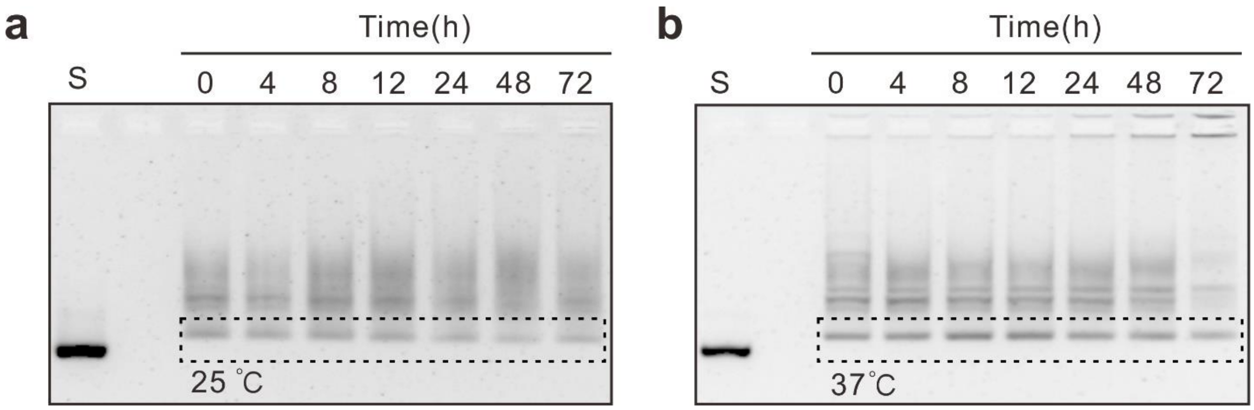

Supplementary Fig. 7. Agarose gel electrophoresis analysis of Dor-pMHCs after incubation in PBS at **(a)** 25 °C and **(b)** 37 °C for different times (0, 4, 8, 12, 24, 48, 72 h). S indicates the M13mp18 scaffold. The rectangle indicates bands of Dor-pMHCs products. Source data are provided as a Source Data file.

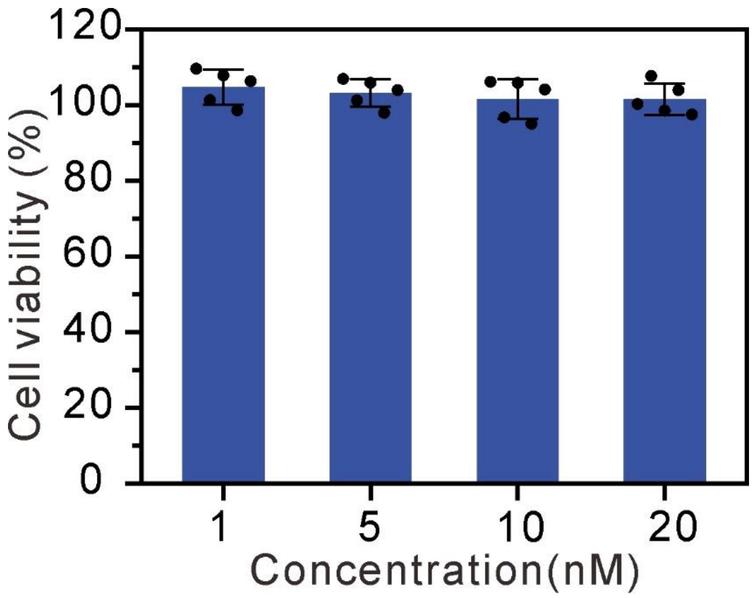

Supplementary Fig. 8. Cytotoxicity assessment of different concentrations of Dor-pMHCs to spleen cells after 12 h incubation by MTT assay. Data represent the mean ± s.d. from n=5 independent experiments. Source data are provided as a source data file.

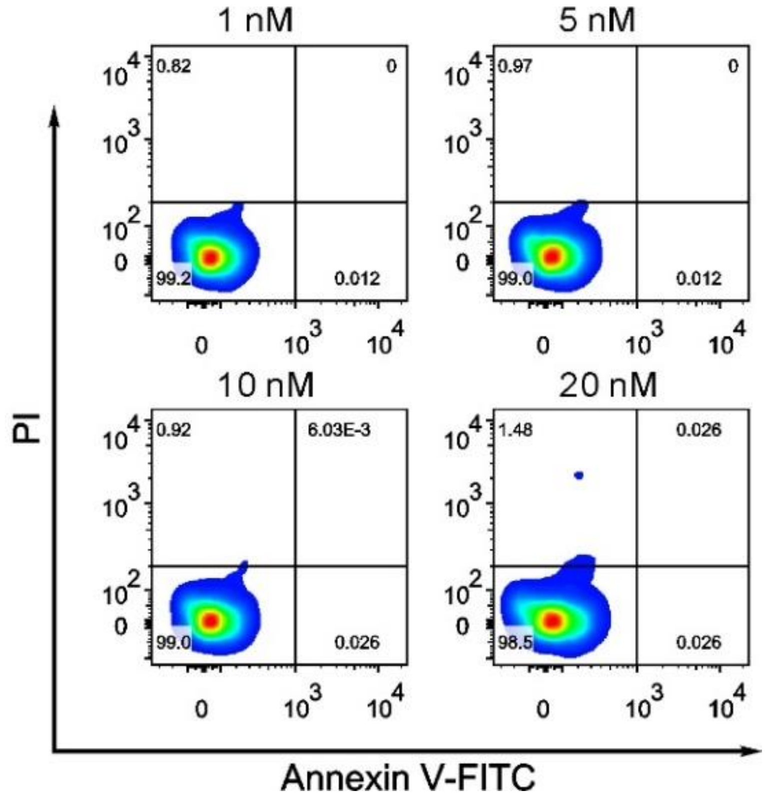

Supplementary Fig. 9. Flow cytometric analysis of splenocytes stained by the Annexin V-FITC/PI after incubation at different concentrations (1 nM, 5 nM, 10 nM, and 20 nM) of Dor-pMHCs for 12 h.

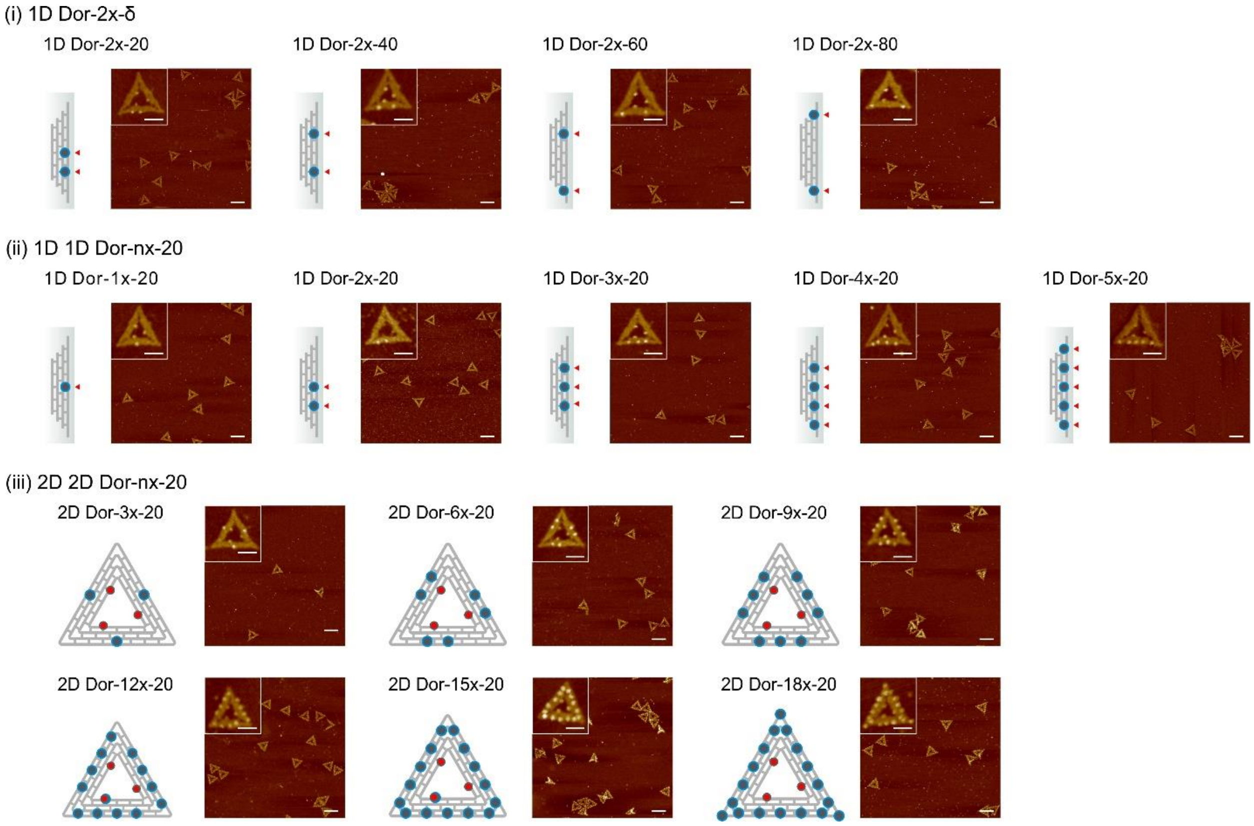

Supplementary Fig. 10. Scheme and AFM images of three series of PE-labelled Dor-pMHCs, including (i) 1D Dor-2x-δ, (ii) 1D Dor-nx-20, and (iii) 2D Dor-nx-20. Scale bar: 100 nm. n indicates the number of SA binding site, δ indicates the inter-pMHC spacing on each edge.

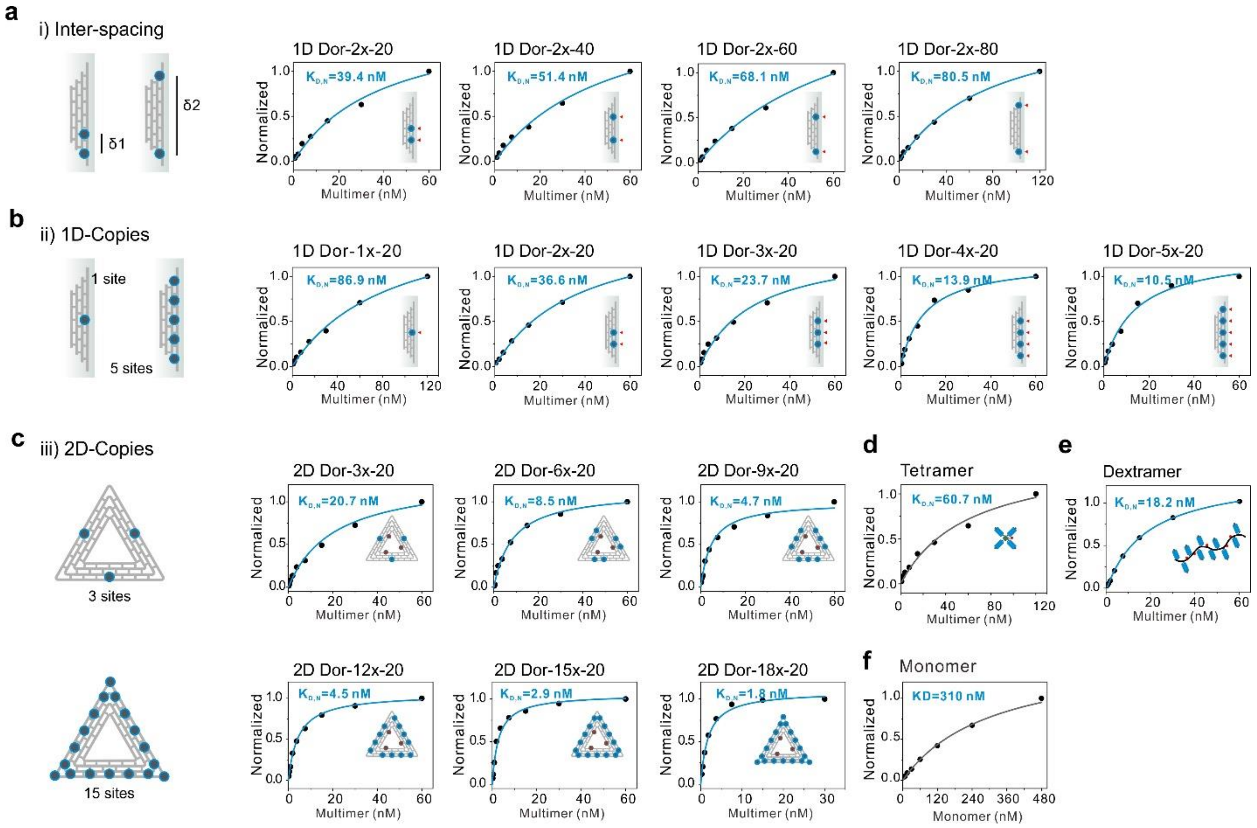

Supplementary Fig. 11. Determination of binding avidity of **(a)** 1D Dor-2x-δ, **(b)** 1D Dor-nx-20, **(c)** 2D Dor-nx-20, **(d)** tetramer, **(e)** dextramer, and **(f)** monomer towards OT-1 naive T cells at 4 °C. The concentration of pMHC multimers used ranged from 0 to 120 nM. The apparent dissociation constants ($K_{D,N}$) were given. Source data are provided as a source data file.

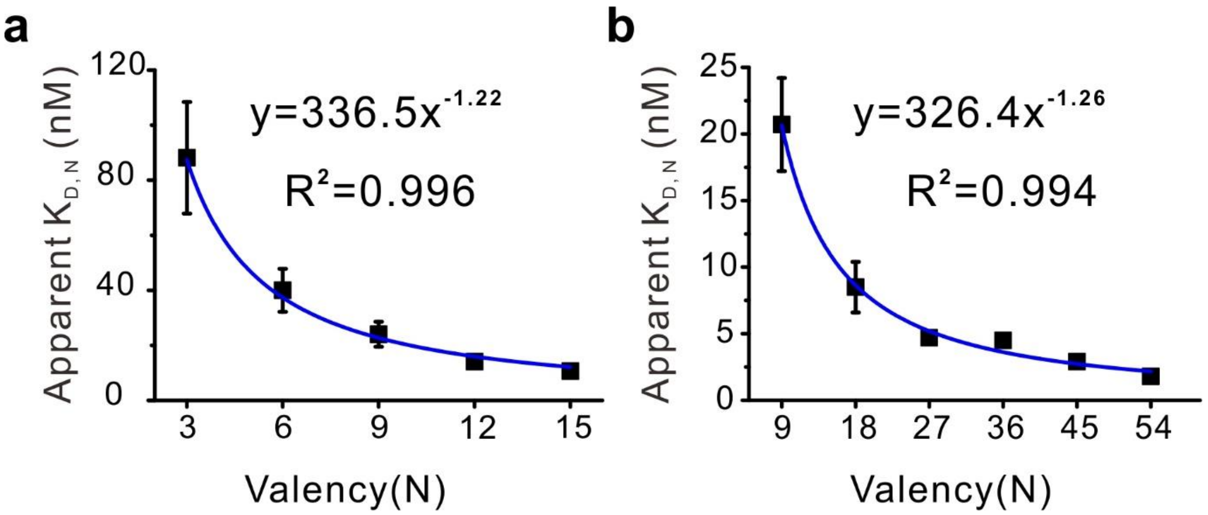

Supplementary Fig. 12. Summary of binding avidity apparent $K_{D,N}$ of **(a)** 1D Dor-nx-20 and **(b)** 2D Dor-nx-20 versus their corresponding pMHC valency. The apparent $K_{D,N}$ was plotted against pMHC valency, and the correlation was fit to a power regression curve. Apparent dissociation constants were given in Table S1. Data represent the mean ± s.d. from n=3 independent experiments. Source data are provided as a source data file.

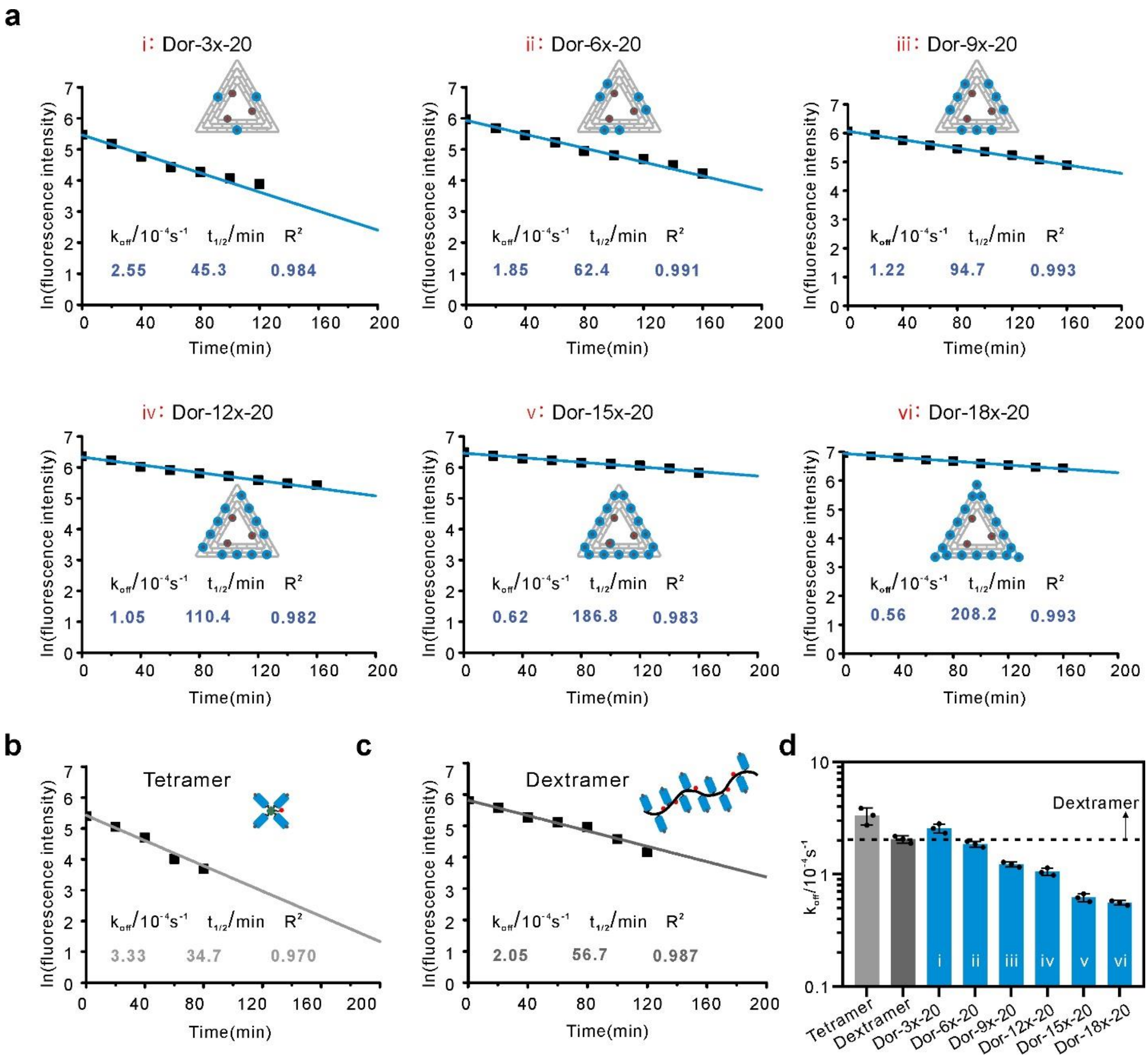

Supplementary Fig. 13. Detection of dissociation kinetics of **(a)** 2D Dor-nx-20, **(b)** tetramer, and **(c)** dextramer binding to OT-1 naive T cells (1×10^6/mL) at 4 °C using flow cytometry in the presence of saturating amounts of anti-H-$2K^b$ MHC antibody. The concentration of pMHC multimers used was 3 nM. Values of dissociation rates (k_{off}) and half-lives ($t_{1/2}$) were given in Table S2. **(d)** Summary of k_{off} of 2D Dor-nx-20, tetramer and dextramer. Data represent the mean ± s.d. from n=3 independent experiments. Source data are provided as a source data file.

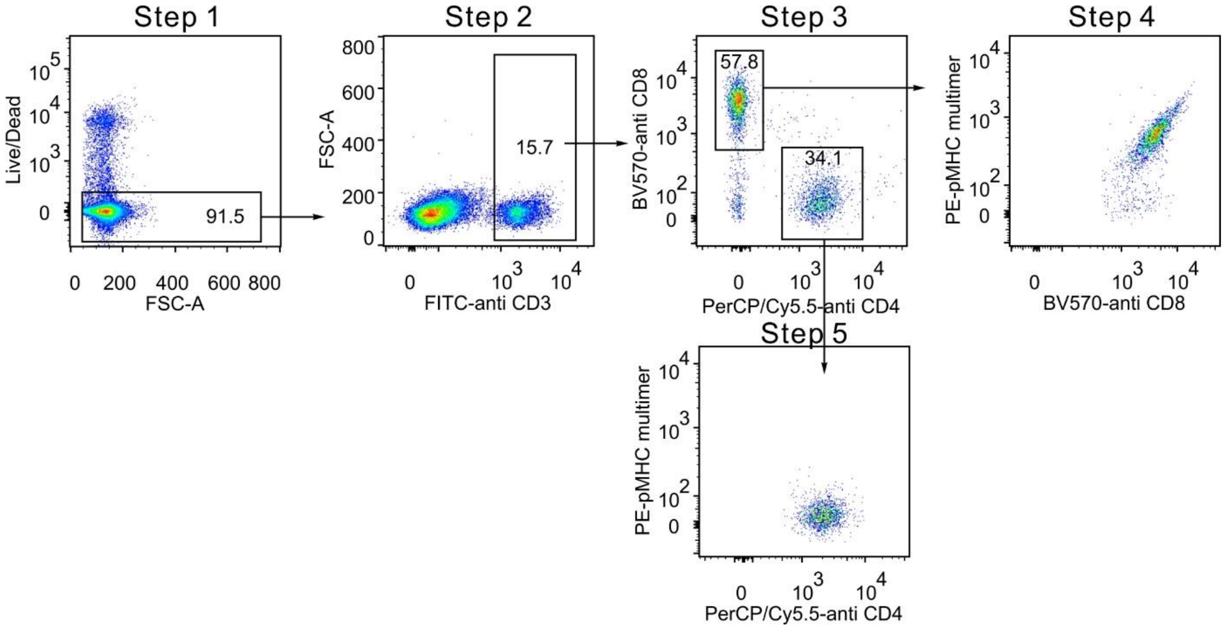

Supplementary Fig. 14. Workflow of antigen-specific staining of OVA-specific CD8$^+$ T cells. Step 1: staining of splenocytes with live/dead aqua stain to sort live cells; Step 2: staining of CD3$^+$ T cells with FITC-anti CD3; Step 3: double-staining of CD3$^+$ T cells with PerCP/Cy5.5-anti CD4 and BV570-anti CD8; Step 4: representative staining of OVA-specific CD8$^+$ T cells by 3 nM PE-pMHC multimer; Step 5: representative background staining of OVA-specific CD4$^+$ T cells by 3 nM PE-pMHC multimer.

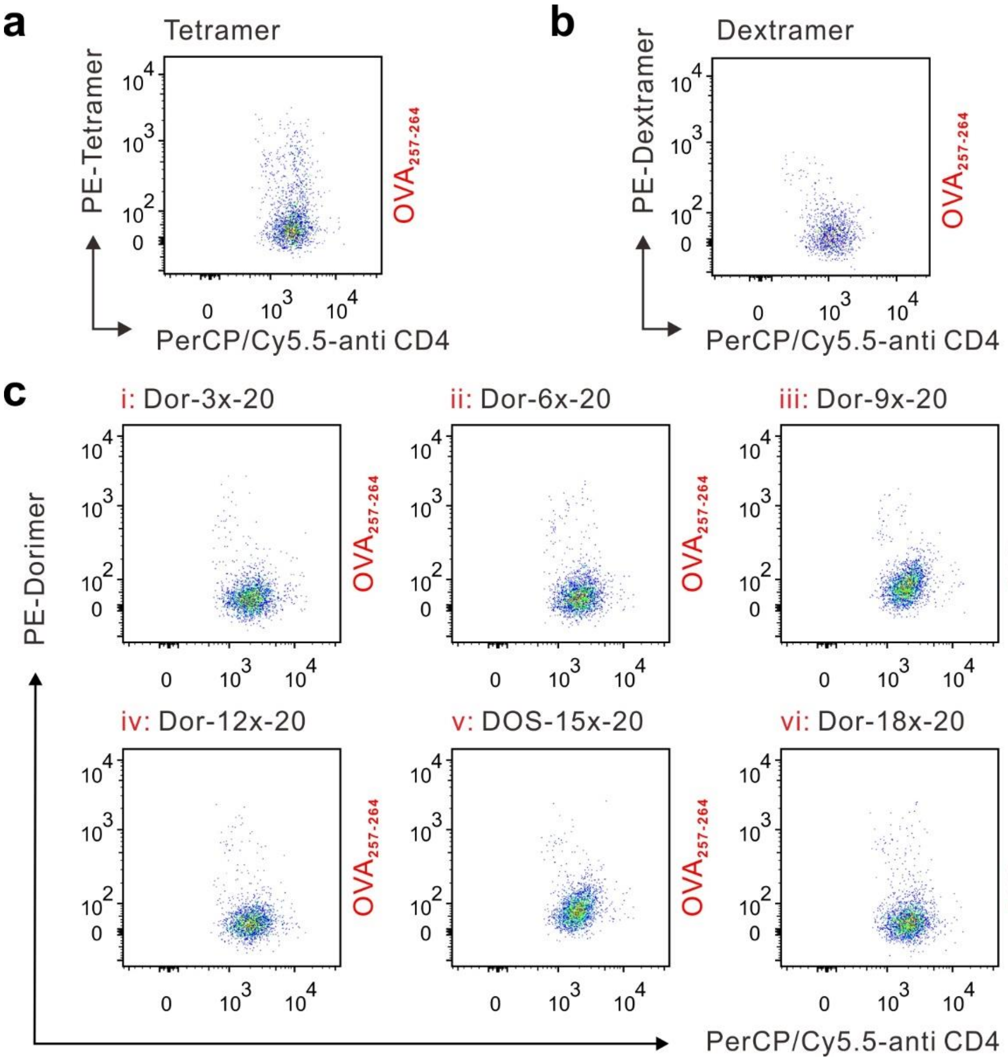

Supplementary Fig. 15. Background staining analysis of $CD4^+$ T cells in splenocytes from OT-1 transgenic mice using **(a)** tetramer, **(b)** dextramer, and **(c)** six types of dorimers (i-vi) at the concentration of 3 nM. pMHC multimers were made using H-$2K^b$ MHC loaded with an OVA peptide.

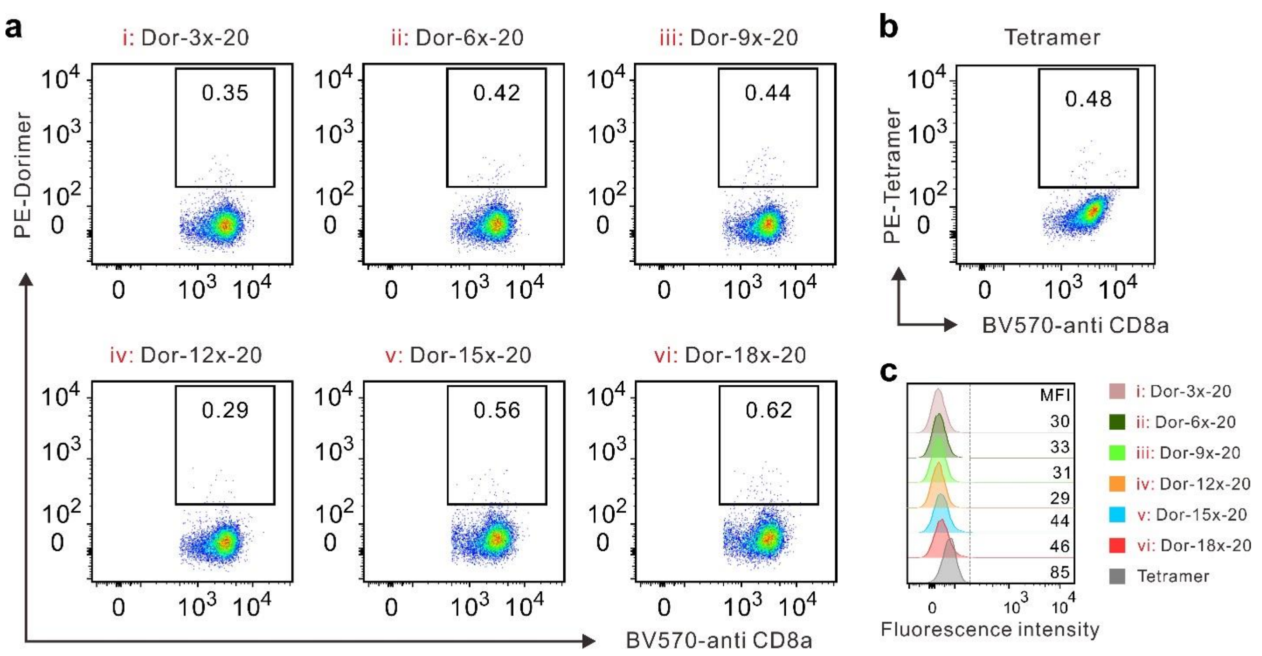

Supplementary Fig. 16. Antigen-specific staining of OVA-specific CD8$^+$ T cells from non-transgenic C57BL/6 mice without ova gene transcription with **(a)** six types of dorimers (i-vi) and **(b)** tetramer at the concentration of 3 nM. pMHC multimers were prepared using H-2K^b MHC loaded with the OVA peptide. **(c)** The mean fluorescence intensity (MFI) of OVA-specific CD8$^+$ T cells stained by dorimers and tetramer. The MFI of staining was shown on the right.

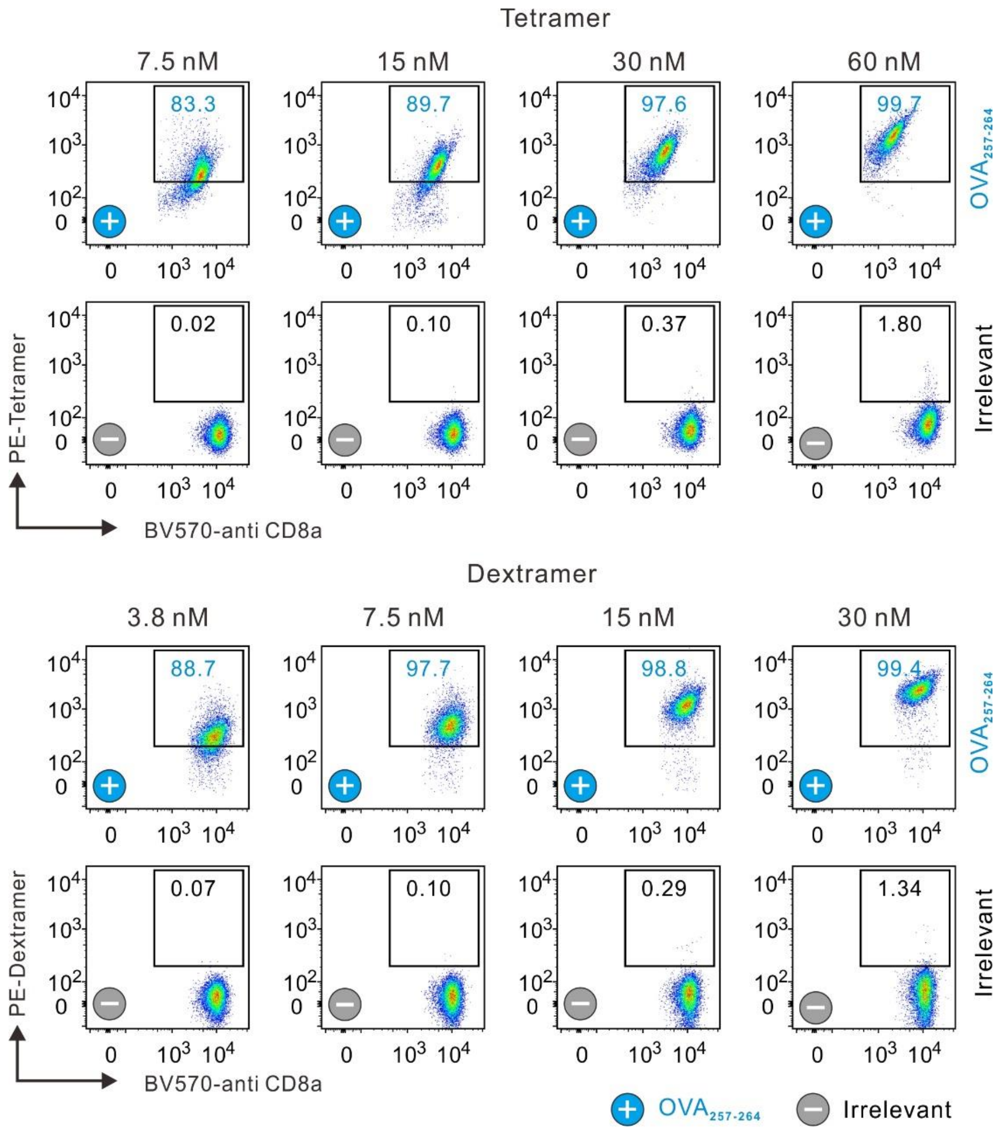

Supplementary Fig. 17. Antigen-specific staining of OVA-specific $CD8^+$ T cells from OT-1 transgenic mice spleen with high concentration of tetramers (7.5 nM, 15 nM, 30 nM, 60 nM) and dextramers (3.8 nM, 7.5 nM, 15 nM, 30 nM). pMHC multimers were made using H-2K^b MHC loaded with the specific OVA peptide (SIINFEKL) **(+)** or an irrelevant peptide (SIYRYYGL) **(–)**.

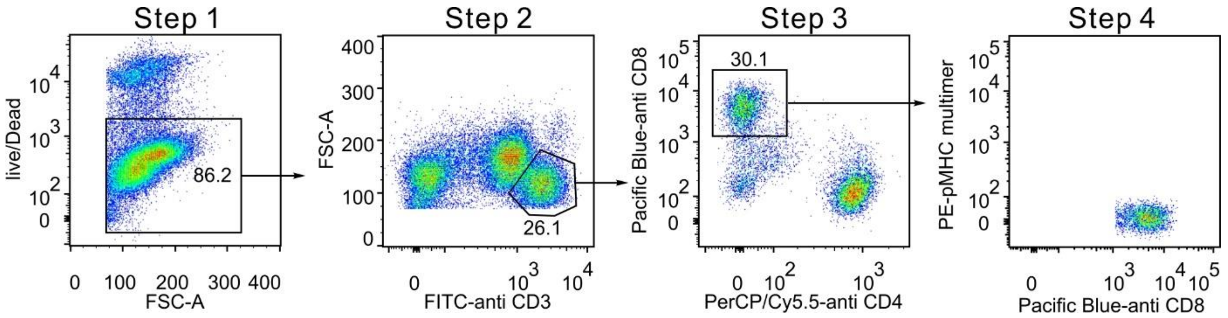

Supplementary Fig. 18. Workflow of antigen-specific staining of CMV-specific CD8$^+$ T cells from PBMCs. Step 1: staining of PBMCs with live/dead aqua stain to sort live cells; Step 2: staining of CD3$^+$ T cells with FITC-anti CD3; Step 3: staining of CD3$^+$ T cells with Pacific Blue-anti CD8; Step 4: representative staining of OVA-specific CD8$^+$ T cells by 3 nM PE-pMHC multimer.

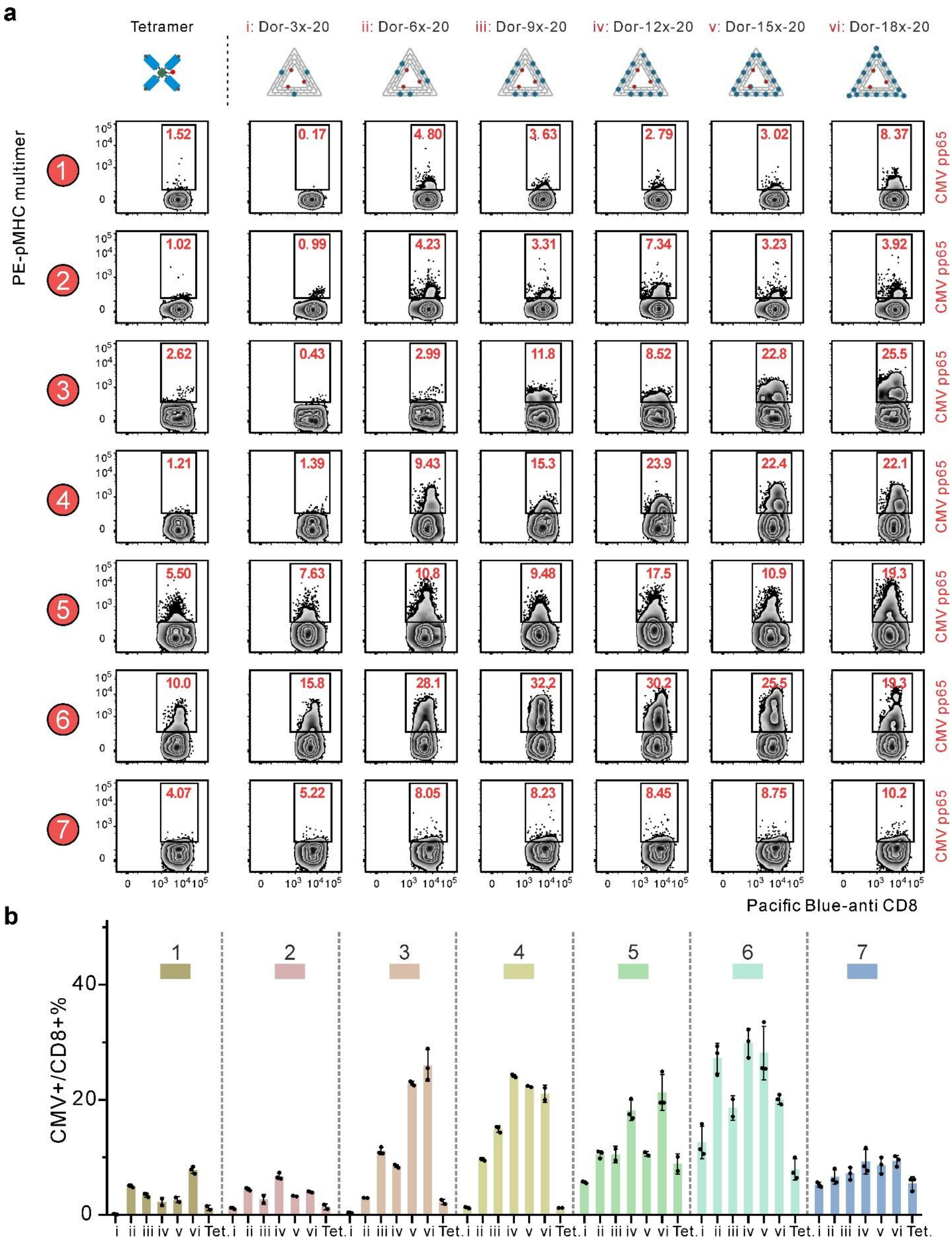

Supplementary Fig. 19. Antigen-specific staining of human CMV-specific CD8$^+$ T cell from CMV positive PBMCs samples by dorimers. **(a)** Representative staining of CMV-specific CD8$^+$ T cell from seven CMV-positive PBMCs samples by six types of dorimers (i-vi) and tetramers at a concentration of 3 nM. pMHC multimers were made using HLA-A2 molecule loaded with the specific CMV peptide (pp65$_{495-503}$, NLVPMVATV). **(b)** Statistical analysis of the percentage of CMV-specific CD8$^+$ T cells from seven PBMCs samples by dorimers and tetramers. Data represent the mean ± s.d. from n=3 independent experiments. Source data are provided as a source data file.

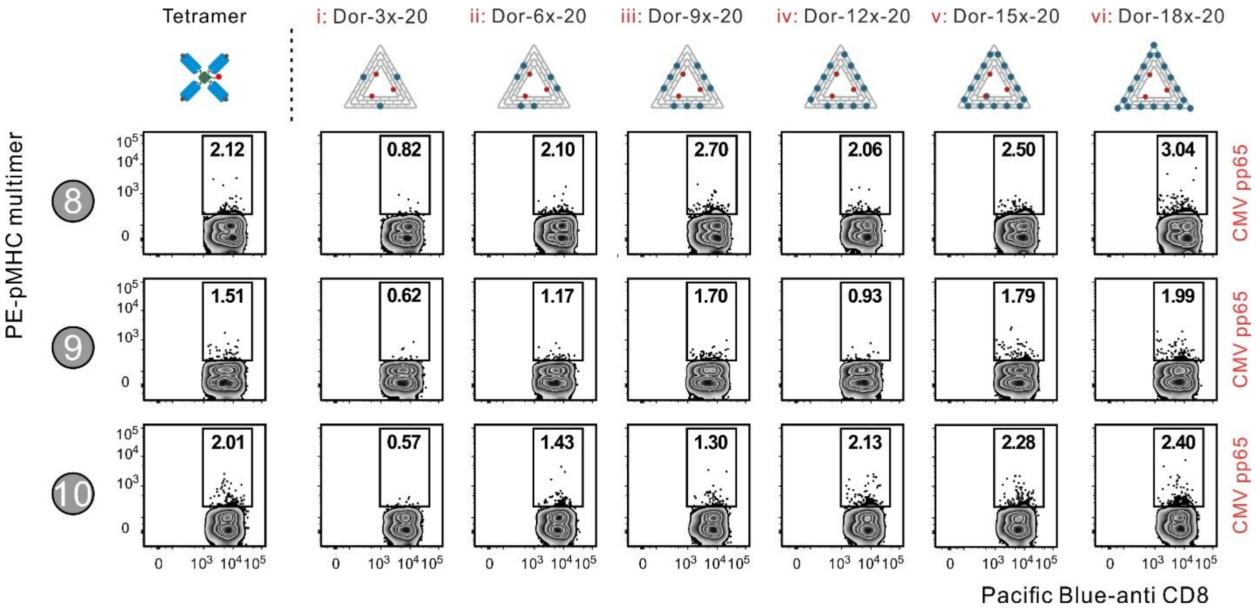

Supplementary Fig. 20. Antigen-specific staining of human CMV-specific CD8$^+$ T cell from CMV negative PBMCs samples by dorimers. Representative staining of CMV-specific CD8$^+$ T cell from three CMV negative PBMCs samples by six types of dorimers (i-vi) and tetramer at 3 nM. pMHC multimers were made using HLA-A2 loaded with the specific CMV peptide (pp65$_{495-503}$, NLVPMVATV). The percentages of the identified CMV-specific CD8$^+$ T cells were given in each panel.

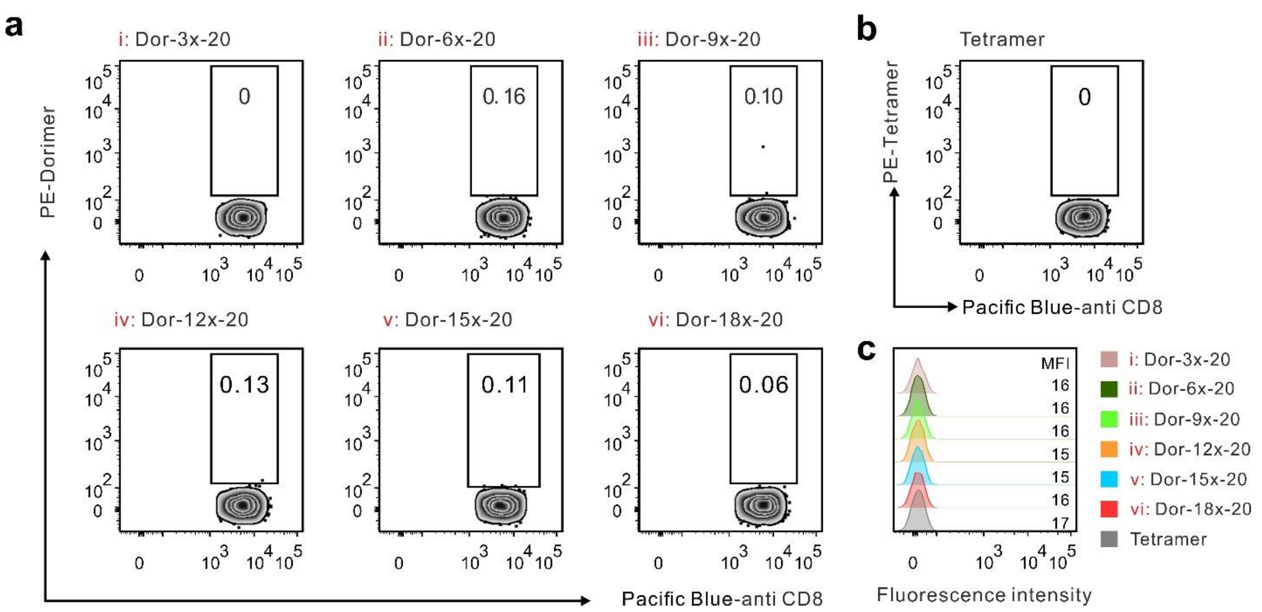

Supplementary Fig. 21. Nonspecific staining of CMV-specific CD8$^+$ T cells by **(a)** six types of dorimers (i-vi) and **(b)** tetramers at 3 nM. pMHC multimers were made using HLA-A2 molecule loaded with the irrelevant HIV peptide (SLYNTVATL). **(c)** The mean fluorescence intensity (MFI) of CD8$^+$ T cells by six types of dorimers (i-vi) and tetramer. The MFI of staining was shown on the right.

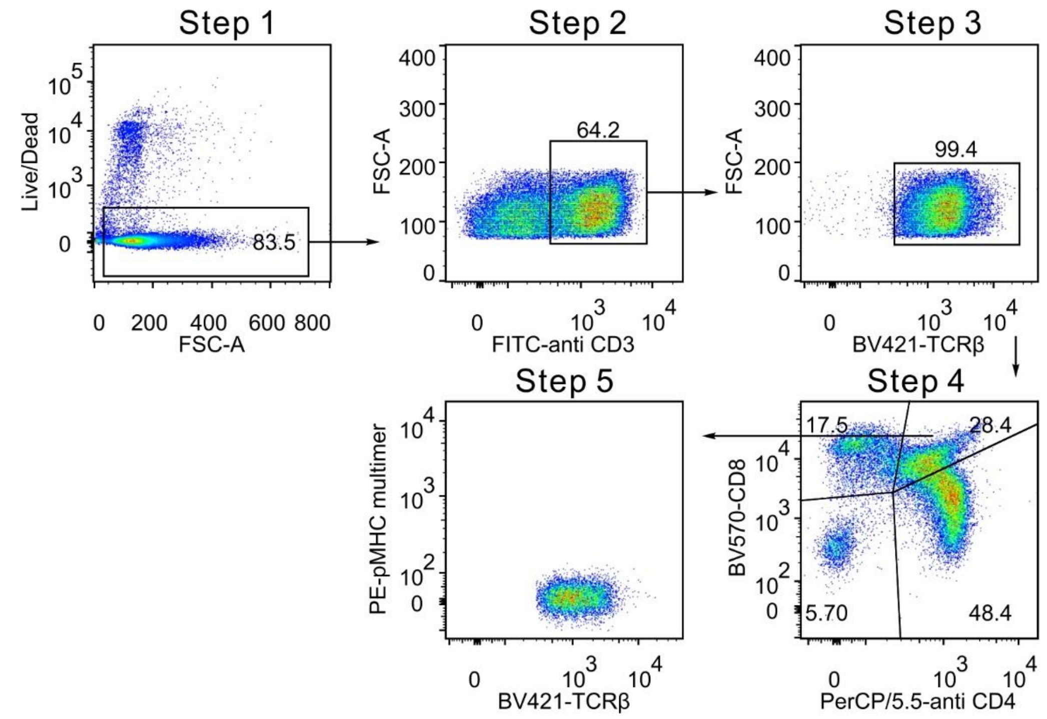

Supplementary Fig. 22. Workflow of antigen-specific staining of $CD4^{+}CD8^{+}$ double-positive thymocytes of OT-1 transgenic mice. Step 1: staining of thymocytes with live/dead aqua stain to sort live cells; Step 2: staining of $CD3^{+}$ T cells with FITC-anti CD3; Step3 and step 4: double-staining of $CD3^{+}$ T cells with PerCP/Cy5.5-anti CD4 and BV570-anti CD8; Step 5: representative staining of $CD4^{+}CD8^{+}$ double-positive T cells by 1 nM PE-pMHC multimer.

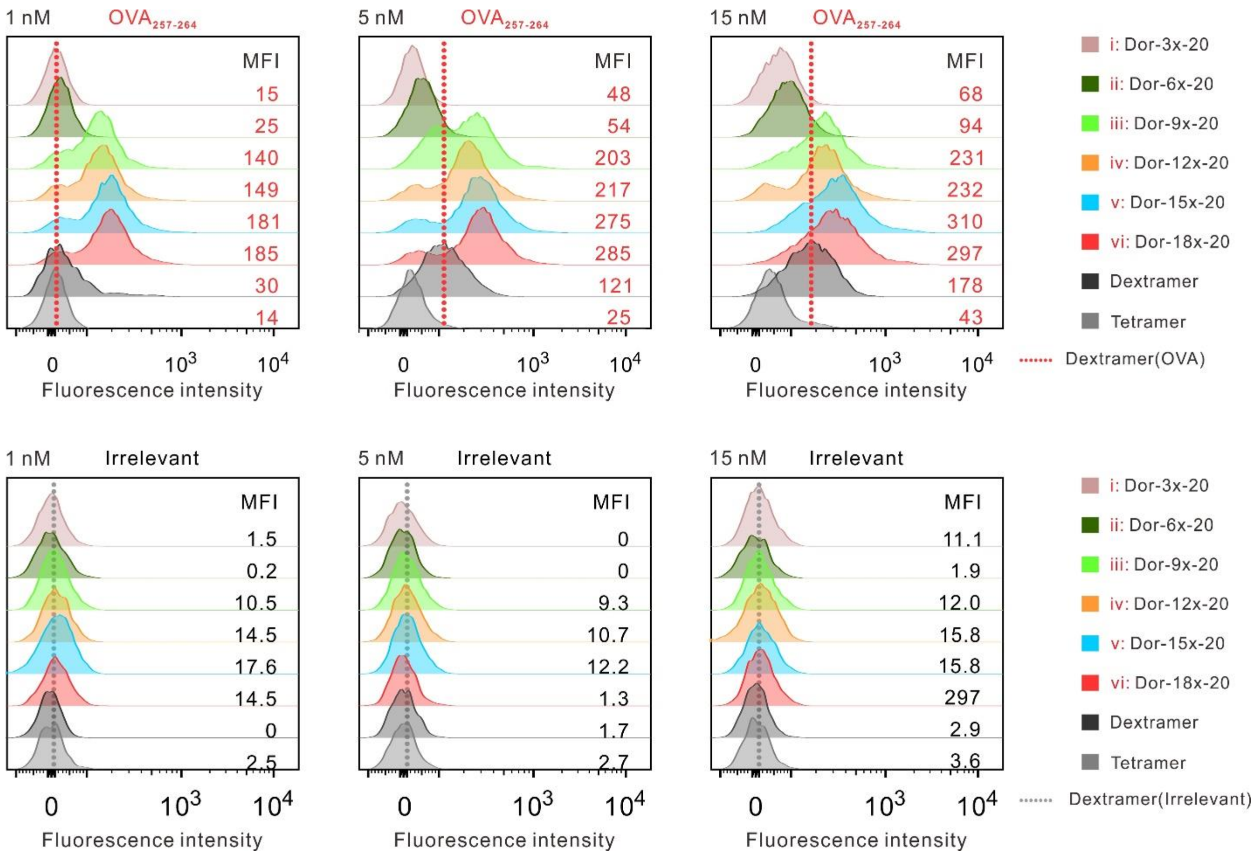

Supplementary Fig. 23. The mean fluorescence intensity (MFI) of OVA-specific $CD4^{+}CD8^{+}$ T cells from OT-1 transgenic mice by different concentrations of dorimers, tetramer and dextramer. The multimers were made using H-$2K^{b}$ MHC loaded with the specific OVA peptide or an irrelevant peptide. The MFI of staining was shown on the right.

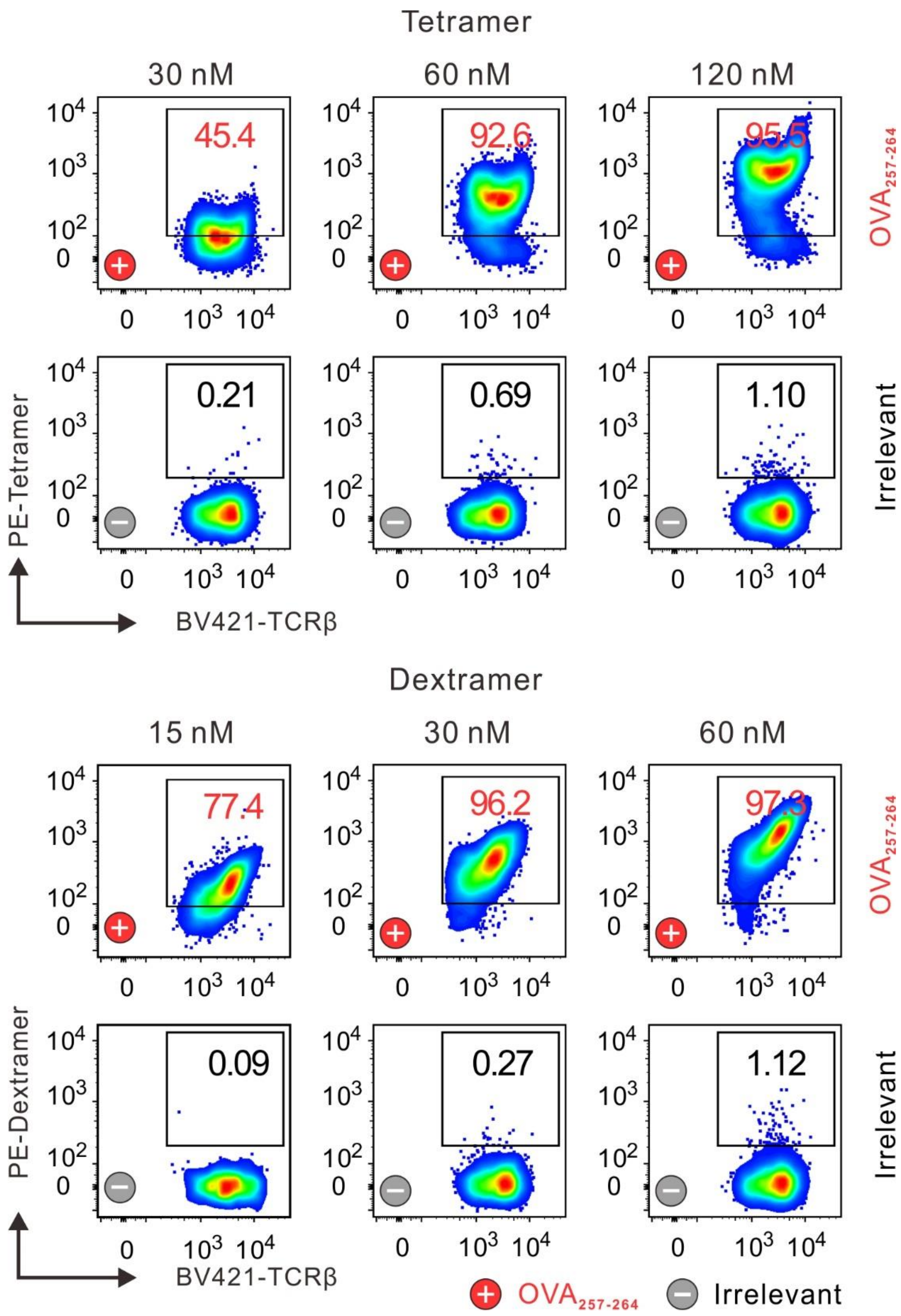

Supplementary Fig. 24. Antigen-specific staining of OVA-specific $CD4^{+}CD8^{+}$ T cells from OT-1 transgenic mice thymus with high concentration of tetramers (30 nM, 60 nM, 120 nM) and dextramers (15 nM, 30 nM, 60 nM). pMHC multimers were made using H-2K^b MHC loaded with the specific OVA peptide (SIINFEKL) **(+)** or an irrelevant peptide (SIYRYYGL) **(–)**.

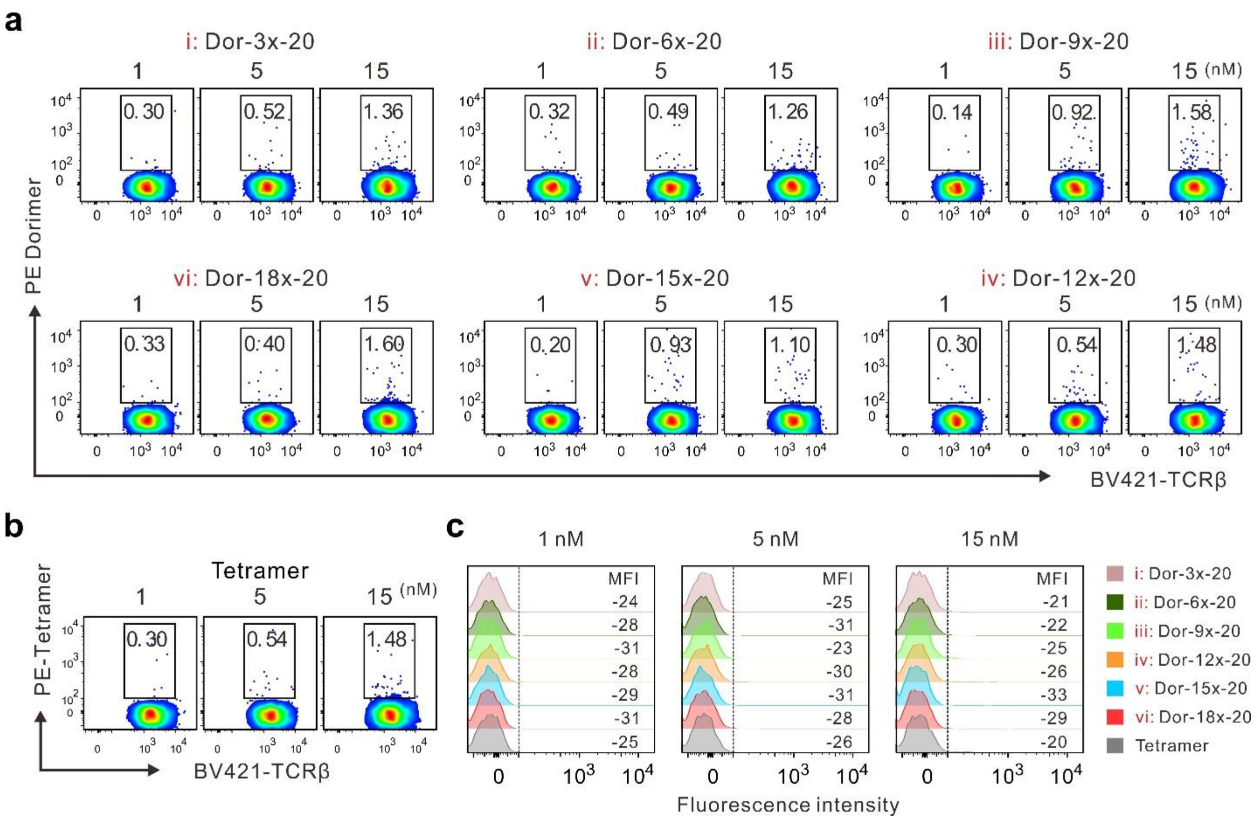

Supplementary Fig. 25. Antigen-specific staining of OVA-specific $CD4^+CD8^+$ double-positive thymocytes from non-transgenic C57BL/6 mice without ova gene transcription by **(a)** six types of dorimers (i-vi) and **(b)** tetramer. pMHC multimers were made using H-2K^b MHC loaded with the OVA peptide. **(c)** The mean fluorescence intensity (MFI) of OVA-specific $CD4^+CD8^+$ double-positive thymocytes by six types of dorimers (i-vi) and tetramer. The MFI of staining was shown on the right.

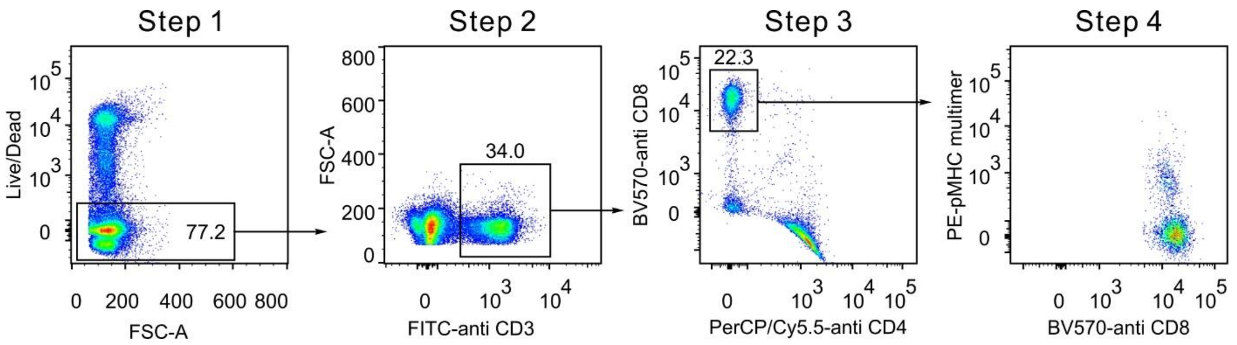

Supplementary Fig. 26. Workflow of antigen-specific staining of autoimmune T cells. Step 1: staining of splenocytes with live/dead aqua stain to sort live cells; Step 2: staining of $CD3^+$ T cells with FITC-anti CD3; Step 3; staining of $CD3^+$ T cells with BV570-antiCD8; Step 4: Representative staining of InsB-specific $CD8^+$ T cells by 10 nM PE-pMHC multimer.

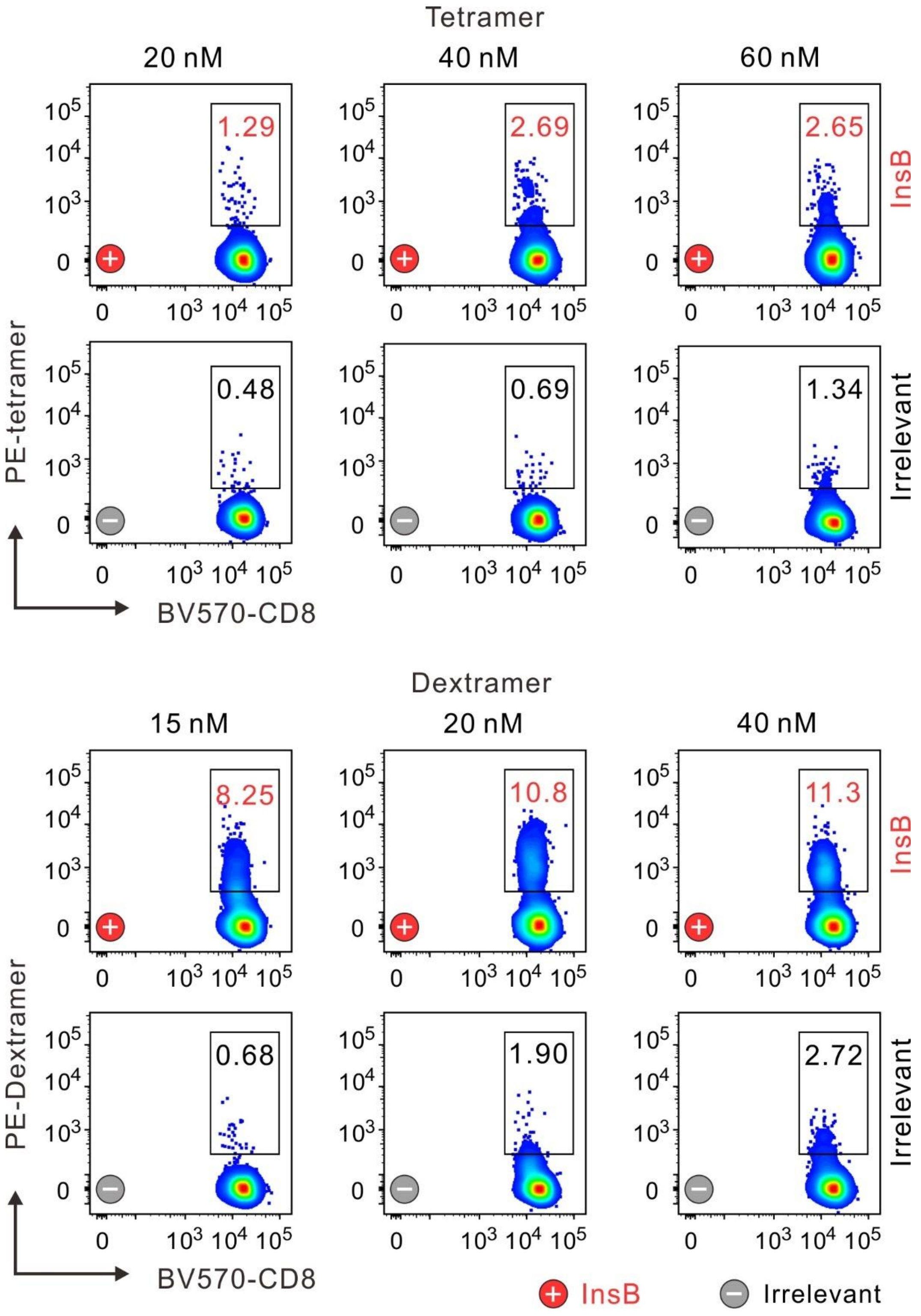

Supplementary Fig. 27. Antigen-specific staining of autoimmune T cells from NOD mice with high concentration of tetramers (20 nM, 40 nM, 60 nM) and dextramers (15 nM, 20 nM, 40 nM). pMHC multimers were made using H-$2K^d$ MHC loaded with the specific InsB peptide (LYLVCGERL) (+) or an irrelevant TUM peptide (KYQAVTTTL).

Supplementary Table 1. Summary of the apparent $K_{D,N}$, the enhancement parameter, and the cooperativity parameter of multimers.

Multimers	$K_{D,N}$ nM	$1/\beta$	α
Monomer	310	1	1
1D Dor-2x-20	39.4	7.9	1.1×10^{-1}
1D Dor-2x-40	51.4	6.0	1.1×10^{-1}
1D Dor-2x-60	68.1	4.6	1.2×10^{-1}
1D Dor-2x-80	80.5	3.9	1.3×10^{-1}
1D Dor-1x-20	86.9	3.6	2.6×10^{-1}
1D Dor-2x-20	36.6	8.5	1.0×10^{-1}
1D Dor-3x-20	23.7	13.1	6.1×10^{-2}
1D Dor-4x-20	13.9	22.3	3.8×10^{-2}
1D Dor-5x-20	10.5	29.5	2.7×10^{-2}
2D Dor-3x-20	20.7	15.0	5.9×10^{-2}
2D Dor-6x-20	8.5	36.5	3.1×10^{-2}
2D Dor-9x-20	4.7	66.0	1.0×10^{-2}
2D Dor-12x-20	4.5	68.9	7.3×10^{-3}
2D Dor-15x-20	2.9	106.9	4.1×10^{-3}
2D Dor-18x-20	1.8	172.2	1.9×10^{-3}
Tetramer	60.7	5.1	1.7×10^{-1}
Dextramer	18.2	17.1	4.2×10^{-2}

Supplementary Table 2. Summary of the off-rate k_{off} and $t_{1/2}$ of 2D kinetics of TCR and multimers interaction.

Multimers	k_{off} $10^{-4}s^{-1}$	$t_{1/2}$ min	R^2
2D Dor-3x-20	2.55	45.3	0.984
2D Dor-6x-20	1.85	62.4	0.991
2D Dor-9x-20	1.22	94.7	0.993
2D Dor-12x-20	1.05	110.4	0.982
2D Dor-15x-20	0.62	186.8	0.983
2D Dor-18x-20	0.56	208.2	0.993
Tetramer	3.33	34.7	0.970
Dextramer	2.05	56.7	0.987

Supplementary Table 3. Detection of OVA-specific $CD8^+$ T cells in spleen from OT-1 mice by multimers at 3 nM.

Multimers	OVA^+CD8^+ %	SIY^+CD8^+ %
Dor-3x-20	67.9	0.01
Dor-6x-20	90.0	0.02
Dor-9x-20	93.7	0.02
Dor-12x-20	94.4	0.06
Dor-15x-20	95.7	0.02
Dor-18x-20	99.3	0.06
Tetramer	60.4	0.01
Dextramer	73.1	0.07

Supplementary Table 4. Detection of OVA-specific $CD8^+$ T cells in spleen from OT-1 mice by tetramer and dextramer at high concentrations.

Concentration (nM)	Tetramer		Dextramer	
	OVA^+CD8^+ %	SIY^+CD8^+ %	OVA^+CD8^+ %	SIY^+CD8^+ %
3.8	/	/	88.7	0.07
7.5	83.3	0.02	97.7	0.10
15	89.7	0.10	98.8	0.29
30	97.6	0.37	99.4	1.34
60	99.7	1.80	/	/

Supplementary Table 5. The comparison of pMHC multimers with equivalent pMHC concentration in both staining efficiency and background for identifying OVA-specific $CD8^+$ T cell from OT-1 mice spleen.

pMHC concentration (nM)	Dorimers OVA$^+$ %^{a} / SIY$^+$ %^{b}	Tetramer OVA$^+$ %^{a} / SIY$^+$ %^{b}	Dextramer OVA$^+$ %^{a} / SIY$^+$ %^{b}
27	Dor-3x-20: 67.9 / 0.01	-	-
30	-	83.3 / 0.02	-
36	-	-	73.1 / 0.07
45	-	-	88.7 / 0.07
54	Dor-6x-20: 90.0 / 0.02 *****	-	-
60	-	89.7 / 0.10	-
81	Dor-9x-20: 93.7 / 0.02 *****	-	-
90	-	-	97.7 / 0.10
108	Dor-12x-20: 94.4 / 0.06 *****	-	-
120	-	97.6 / 0.37	-
135	Dor-15x-20: 95.7 / 0.02 *****	-	-
162	Dor-18x-20: 99.3 / 0.06 *****	-	-
180	-	-	98.8 / 0.29
240	-	99.7 / 1.80	-

a: the percentage of specific binding

b: the percentage of nonspecific binding

* Dorimer is better than tetramer and dextramer with equivalent pMHC concentration in staining efficiency or background. For example, Dor-6x-20 with equivalent pMHC concentration of 54 nM has comparable frequency of specific T cells and less nonspecific binding than tetramer with equivalent pMHC concentration of 60 nM (Dor-6x-20: 90.0 / 0.02 *vs* Tetramer: 89.7 / 0.10).

Supplementary Table 6. Detection of double-positive $CD4^+CD8^+$ T cells in the thymus from OT-1 mice by multimers.

Multimers	$OVA^+CD4^+CD8^+$ %			$SIY^+CD4^+CD8^+$ %		
	1	5	15	1	5	15
Dor-3x-20	0.44	9.76	22.3	0.00	0.00	0.00
Dor-6x-20	1.64	12.0	42.7	0.00	0.09	0.09
Dor-9x-20	67.9	74.3	81.5	0.00	0.04	0.05
Dor-12x-20	70.7	81.1	84.5	0.00	0.06	0.06
Dor-15x-20	77.6	83.7	91.7	0.04	0.06	0.07
Dor-18x-20	78.8	84.1	91.1	0.07	0.09	0.04
Tetramer	0.27	4.71	12.4	0.00	0.00	0.00
Dextramer	15.1	57.8	77.4	0.00	0.09	0.09

Supplementary Table 7. Detection of double-positive $CD4^+CD8^+$ T cells in the thymus from OT-1 mice by tetramer and dextramer at high concentrations.

Concentration (nM)	Tetramer		Dextramer	
	OVA^+ $CD4^+CD8^+$ %	SIY^+ $CD4^+CD8^+$ %	OVA^+ $CD4^+CD8^+$ %	SIY^+ $CD4^+CD8^+$ %
15	/	/	77.4	0.09
30	45.4	0.21	96.2	0.27
60	92.6	0.69	97.3	1.12
120	95.5	1.10	/	/

Supplementary Table 8. The comparison of pMHC multimers with equivalent pMHC concentration in both staining efficiency and background for identifying double-positive $CD4^{+}CD8^{+}$ T cells from OT-1 mice thymus.

pMHC concentration (nM)	Dorimers OVA^{+} % ^{a} / SIY^{+} % ^{b}	Tetramer OVA^{+} % ^{a} / SIY^{+} % ^{b}	Dextramer OVA^{+} % ^{a} / SIY^{+} % ^{b}
9	Dor-3x-20: 0.44 / 0.00	-	-
12	-	-	15.1 / 0.00
18	Dor-6x-20: 1.64 / 0.00	-	-
20	-	4.71 / 0.00	-
27	Dor-9x-20: 67.9 / 0.00 *	-	-
36	Dor-12x-20: 70.7 / 0.00 *	-	-
45	Dor-15x-20: 77.6 / 0.04 *<br>Dor-3x-20: 9.76 / 0.00	-	-
54	Dor-18x-20: 78.8 / 0.07 *	-	-
60	-	12.4 / 0.00	57.8 / 0.09
90	Dor-6x-20: 12.0 / 0.09	-	-
120	-	45.4 / 0.21	-
135	Dor-3x-20: 22.3 / 0.00<br>Dor-9x-20: 74.3 / 0.04	-	-
180	Dor-12x-20: 81.1 / 0.06 *	-	77.4 / 0.09
225	Dor-15x-20: 83.7 / 0.06	-	-
240	-	92.6 / 0.69	-
270	Dor-6x-20 42.7 / 0.09<br>Dor-18x-20: 84.1 / 0.09	-	-
360	-	-	96.2 / 0.27
405	Dor-9x-20 81.5 / 0.05	-	-
480	-	95.5 / 1.10	-
540	Dor-12x-20 84.5 / 0.06	-	-
675	Dor-15x-20 91.7/ 0.07 *	-	-
720	-	-	97.3 / 1.12
810	Dor-18x-20 91.1/ 0.04 *	-	-

a: the percentage of specific binding

b: the percentage of nonspecific binding

* Dorimer is better than tetramer and dextramer with equivalent pMHC concentration in staining efficiency or background. For example, Dor-9x-20 with equivalent pMHC concentration of 54 nM has a higher frequency of specific T cells and less nonspecific binding both than tetramer and dextramer with equivalent pMHC concentration of 60 nM (Dor-9x-20: 67.9 / 0.00 *vs* Tetramer: 12.4 / 0.00 *vs* Dextramer: 57.8 / 0.09). Note that Dor-15x-20 with equivalent pMHC concentration of 675 nM has slightly lower frequency of specific T cells but much less nonspecific binding dextramer with equivalent pMHC concentration of 720 nM (Dor-15x-20: 91.7 / 0.07 *vs* Dextramer: 97.3 / 1.12).

Supplementary Table 9. Detection of autoimmune $CD8^+$ T cells in spleen from NOD mice by multimers at 10 nM.

Multimers	$InsB^+CD8^+$ %	TUM^+CD8^+ %
Dor-3x-20	2.63	0.00
Dor-6x-20	12.4	0.00
Dor-9x-20	13.0	0.00
Dor-12x-20	14.5	0.06
Dor-15x-20	16.4	0.07
Dor-18x-20	17.4	0.10
Tetramer	0.97	0.00
Dextramer	7.80	0.07

Supplementary Table 10. Detection of autoimmune $CD8^+$ T cells in spleen from NOD mice by tetramer and dextramer at high concentrations.

Concentration (nM)	Tetramer		Dextramer	
	$InsB^+CD8^+$ %	TUM^+CD8^+ %	$InsB^+CD8^+$ %	TUM^+CD8^+ %
15	/	/	8.25	0.68
20	1.29	0.48	10.8	1.90
40	2.69	0.69	11.3	2.72
60	2.65	1.34	/	/

Supplementary Table 11. The comparison of pMHC multimers with equivalent pMHC concentration in both staining efficiency and background for identifying autoimmune $CD8^+$ T cells from NOD mice spleen.

pMHC concentration (nM)	Dorimers InsB$^+$ % [a] / TUM$^+$ % [b]	Tetramer InsB$^+$ % [a] / TUM$^+$ % [b]	Dextramer InsB$^+$ % [a] / TUM$^+$ % [b]
80		1.29 / 0.48	
90	Dor-3x-20: 2.63 / 0.00		
120			7.80 / 0.07
160		2.69 / 0.69	
180	Dor-6x-20: 12.4 / 0.00 *		8.25/ 0.68
240		2.65 / 1.34	10.8 / 1.90
270	Dor-9x-20: 13.0 / 0.00 *		
360	Dor-12x-20: 14.5 / 0.06 *		
450	Dor-15x-20: 16.4 /0.07 *		
480			11.3 / 2.72
540	Dor-18x-20: 17.4 / 0.10 *		
720			

a: the percentage of specific binding

b: the percentage of nonspecific binding

* Dorimer is better than tetramer and dextramer with equivalent pMHC concentration in staining efficiency or background. For example, Dor-6x-20 with equivalent pMHC concentration of 180 nM has a higher frequency of specific T cells and less nonspecific binding than tetramer with equivalent pMHC concentration of 240 nM and dextramer with equivalent pMHC concentration of 180 nM (Dor-6x-20: 12.4 / 0.00 *vs* Tetramer: 2.65 / 1.34 *vs* Detramer: 8.25/ 0.68).
