# Supplementary material for: Nanoscale organization of two-dimensional multimeric pMHC reagents with DNA origami for CD8+ T cell detection
Source: Nat Commun. 2022 Jul 7;13:3916. doi: 10.1038/s41467-022-31684-8 (PMC9263106; doi:10.1038/s41467-022-31684-8)
Supplement: Supplementary file 2 — Description of Additional Supplementary Files [file 41467_2022_31684_MOESM2_ESM.docx]

**Description of Additional Supplementary Files**

**Title:** Supplementary Data 1.

**Description:** The sequences of DNA used in this work, including biotinylate DNA, overhangs DNA, and other staple DNA.
