# Supplementary material for: Nanoscale organization of two-dimensional multimeric pMHC reagents with DNA origami for CD8+ T cell detection
Source: Nat Commun. 2022 Jul 7;13:3916. doi: 10.1038/s41467-022-31684-8 (PMC9263106; doi:10.1038/s41467-022-31684-8)
Supplement: Supplementary file 4 — Reporting Summary [file 41467_2022_31684_MOESM4_ESM.pdf]

nature research

Corresponding author(s): Hao Pei

Last updated by author(s): May 9, 2022

Reporting Summary

Nature Research wishes to improve the reproducibility of the work that we publish. This form provides structure for consistency and transparency in reporting. For further information on Nature Research policies, see our Editorial Policies and the Editorial Policy Checklist.

Statistics

For all statistical analyses, confirm that the following items are present in the figure legend, table legend, main text, or Methods section.

n/a	Confirmed	
☐	☒	The exact sample size (n) for each experimental group/condition, given as a discrete number and unit of measurement
☐	☒	A statement on whether measurements were taken from distinct samples or whether the same sample was measured repeatedly
☐	☒	The statistical test(s) used AND whether they are one- or two-sided<br>*Only common tests should be described solely by name; describe more complex techniques in the Methods section.*
☒	☐	A description of all covariates tested
☒	☐	A description of any assumptions or corrections, such as tests of normality and adjustment for multiple comparisons
☐	☒	A full description of the statistical parameters including central tendency (e.g. means) or other basic estimates (e.g. regression coefficient) AND variation (e.g. standard deviation) or associated estimates of uncertainty (e.g. confidence intervals)
☐	☒	For null hypothesis testing, the test statistic (e.g. F, t, r) with confidence intervals, effect sizes, degrees of freedom and P value noted<br>*Give P values as exact values whenever suitable.*
☒	☐	For Bayesian analysis, information on the choice of priors and Markov chain Monte Carlo settings
☒	☐	For hierarchical and complex designs, identification of the appropriate level for tests and full reporting of outcomes
☒	☐	Estimates of effect sizes (e.g. Cohen's d, Pearson's r), indicating how they were calculated

Our web collection on statistics for biologists contains articles on many of the points above.

Software and code

Policy information about availability of computer code

Data collection	No software was used.
Data analysis	FlowJo V10 was used to analyze the flow cytometry data. Origin 8.0 Pro was used to analyze the data.

For manuscripts utilizing custom algorithms or software that are central to the research but not yet described in published literature, software must be made available to editors and reviewers. We strongly encourage code deposition in a community repository (e.g. GitHub). See the Nature Research guidelines for submitting code & software for further information.

Data

Policy information about availability of data

All manuscripts must include a data availability statement. This statement should provide the following information, where applicable:

- Accession codes, unique identifiers, or web links for publicly available datasets
- A list of figures that have associated raw data
- A description of any restrictions on data availability

The data generated and analyzed that support the findings within this paper as well as the DNA origami design is available from the corresponding author upon reasonable request to the corresponding author. The main data supporting the results in this study are available within the Article, Supplementary Information or Source data file. Source data are provided within this paper

Field-specific reporting

Please select the one below that is the best fit for your research. If you are not sure, read the appropriate sections before making your selection.

- [x] Life sciences
- [ ] Behavioural & social sciences
- [ ] Ecological, evolutionary & environmental sciences

For a reference copy of the document with all sections, see nature.com/documents/nr-reporting-summary-flat.pdf

Life sciences study design

All studies must disclose on these points even when the disclosure is negative.

Sample size	All biochemical and biological experiments were performed in three replicates or more. Sample sizes were determined on the basis of estimates from preliminary experiments and previous similar work (PNAS, 2016,113,1890-1897; Nat. Biotechnol. 2016, 34, 1037-1045; Nat. Nanotechnol. 2020, 15, 716-723; Front. Immunol. 2018, 9, 1378), so that reasonable statistical analyses could be conducted.
Data exclusions	No data were excluded.
Replication	Experiments were performed multiple times to confirm observations. All AFM images presented in the manuscript and the Supplementary Information are exemplary micrographs and show representative images of many acquired micrographs. All agarose gel electrophoresis experiments were repeated independently multiple times and reliably reproduced the same results. The number of replicates for other experiments is provided in the caption of each figures. All attempts at replication were successful.
Randomization	Unless when restricted by the genotype, mice and cells were randomly divided into the experimental groups by the process of sample preparation.
Blinding	The experimenters were not blinded to the experimental design as the genotype for mice and cells are already known.

Reporting for specific materials, systems and methods

We require information from authors about some types of materials, experimental systems and methods used in many studies. Here, indicate whether each material, system or method listed is relevant to your study. If you are not sure if a list item applies to your research, read the appropriate section before selecting a response.

Materials & experimental systems

n/a	Involved in the study	
[ ]	[x]	Antibodies
[ ]	[x]	Eukaryotic cell lines
[x]	[ ]	Palaeontology and archaeology
[ ]	[x]	Animals and other organisms
[x]	[ ]	Human research participants
[x]	[ ]	Clinical data
[x]	[ ]	Dual use research of concern

Methods

n/a	Involved in the study	
[x]	[ ]	ChIP-seq
[ ]	[x]	Flow cytometry
[x]	[ ]	MRI-based neuroimaging

Antibodies

Antibodies used	FITC anti-mouse CD3 Antibody (Biolegend; cat. #100204; clone 17A2), 1:100 dilution<br>PerCP/Cyanine5.5 anti-mouse CD4 Antibody (Biolegend; cat. #100434; clone GK1.5), 1:100 dilution<br>Brilliant Violet 570™ anti-mouse CD8a Antibody (Biolegend; cat. #100739; clone 53-6.7), 1:100 dilution<br>Purified anti-mouse CD16/32 Antibody (Biolegend; cat. #101301; clone 93), 1:100 dilution<br>Purified anti-mouse/human CD11b Antibody (Biolegend; cat. #101201; clone M1/70), 1:100 dilution<br>Purified anti-mouse CD11c Antibody (Biolegend; cat. #117301; clone N418), 1:100 dilution<br>Purified anti-mouse Ly-6G/Ly-6C (Gr-1) Antibody (Biolegend; cat. #108401; clone RB6-8C5), 1:100 dilution<br>Purified anti-mouse F4/80 Antibody (Biolegend; cat. #123101; clone BM8), 1:100 dilution<br>Brilliant Violet 421™ anti-mouse TCR β chain Antibody (Biolegend; cat. #109229; clone H57-597), 1:100 dilution<br>FITC anti-human CD3 (Biolegend; cat. #317306; clone OKT3), 1:100 dilution<br>PerCP/Cyanine5.5 anti-human CD4 (Biolegend; cat. #317427; clone OKT4), 1:100 dilution<br>Pacific Blue™ anti-human CD8 (Biolegend; cat. #344717; clone SK1), 1:100 dilution<br>Purified anti-human CD19 (Biolegend; cat. #302202; clone HIB19), 1:100 dilution<br>Purified anti-H-2Kb MHC antibody (Biolegend; cat. #114602; clone 28-8-6), 50 μg/mL
Validation	All antibodies were validated as described on the websites of Biolegend.

FITC anti-mouse CD3 Antibody (Biolegend; cat. #100204; clone 17A2)
https://www.biolegend.com/en-us/products/fitc-anti-mouse-cd3-antibody-45

PerCP/Cyanine5.5 anti-mouse CD4 Antibody (Biolegend; cat. #100434; clone GK1.5)
https://www.biolegend.com/en-us/products/percp-cyanine5-5-anti-mouse-cd4-antibody-4220

Brilliant Violet 570™ anti-mouse CD8a Antibody (Biolegend; cat. #100739; clone 53-6.7)
https://www.biolegend.com/en-us/products/brilliant-violet-570-anti-mouse-cd8a-antibody-7377

Purified anti-mouse/human CD11b Antibody (Biolegend; cat. #101201; clone M1/70)
https://www.biolegend.com/en-us/products/purified-anti-mouse-human-cd11b-antibody-351

Purified anti-mouse CD16/32 Antibody (Biolegend; cat. #101301; clone 93)
https://www.biolegend.com/en-us/products/purified-anti-mouse-cd16-32-antibody-190

Purified anti-mouse CD11c Antibody (Biolegend; cat. #117301; clone N418)
https://www.biolegend.com/en-us/products/purified-anti-mouse-cd11c-antibody-1817

Purified anti-mouse Ly-6G/Ly-6C (Gr-1) Antibody (Biolegend; cat. #108401; clone RB6-8C5)
https://www.biolegend.com/en-us/products/purified-anti-mouse-ly-6g-ly-6c-gr-1-antibody-462

Purified anti-mouse F4/80 Antibody (Biolegend; cat. #123101; clone BM8)
https://www.biolegend.com/en-us/products/purified-anti-mouse-f4-80-antibody-4064

Brilliant Violet 421™ anti-mouse TCR β chain Antibody (Biolegend; cat. #109229; clone H57-597)
https://www.biolegend.com/en-us/products/brilliant-violet-421-anti-mouse-tcr-beta-chain-antibody-7251

FITC anti-human CD3 (Biolegend; cat. #317306; clone OKT3)
https://www.biolegend.com/en-us/products/fitc-anti-human-cd3-antibody-3644

PerCP/Cyanine5.5 anti-human CD4 (Biolegend; cat. #317427; clone OKT4)
https://www.biolegend.com/en-us/products/percp-cyanine5-5-anti-human-cd4-antibody-5011

Pacific Blue™ anti-human CD8 (Biolegend; cat. #344717; clone SK1)
https://www.biolegend.com/en-us/products/pacific-blue-anti-human-cd8-antibody-6509

Purified anti-human CD19 (Biolegend; cat. #302202; clone HIB19)
https://www.biolegend.com/en-us/products/purified-anti-human-cd19-antibody-721

Purified anti-H-2Kb MHC antibody (Biolegend; cat. #114602; clone 28-8-6)
https://www.biolegend.com/en-us/products/purified-anti-mouse-h-2k-b-h-2d-b-antibody-1684

Eukaryotic cell lines

Policy information about cell lines

Cell line source(s)	Mouse splenocytes and thymocytes were harvest from C57BL/6 or OT-1 or NOD mice. Frozen human PBMCs (stored in liquid nitrogen before use) from HLA-A2 positive healthy donor that were nonidentifiable were purchased from Miao Tong Biotechnology Co., LTD
Authentication	All cells were authenticated by antibody staining with anti-CD3, anti-CD4, anti-CD8.
Mycoplasma contamination	All cell lines tested negative for mycoplasm.
Commonly misidentified lines (See ICLAC register)	No commonly misidentified lines were used in this study.

Animals and other organisms

Policy information about studies involving animals; ARRIVE guidelines recommended for reporting animal research

Laboratory animals	Female 30-83 mice, 4-8 weeks old were used in this study. The specify the strains of laboratory animals used were C57BL/6 or OT-1 or NOD mice. Male and female mice were used between 4 and 8 weeks of age. C57BL/6 mice and OT-1 mice were on a pure C57BL/6 genetic background. NOD mice were on a pure NOD/ShiLtJGpt genetic background. The mice were housed housed under Specific Pathogen Free (SPF) conditions with a 12 h light/dark cycle at 22 °C (45% relative humidity), and with food and water ad libitum. All mouse experiments were carried out with sex and age matched groups.
Wild animals	The study did not involve wild animals.

Field-collected samples	The study did not involve samples collected from the field.
Ethics oversight	Mice were carried out in accordance with the guidelines for the care and use of laboratory animals approved by the Animal Ethics Committee of East China Normal University.

Note that full information on the approval of the study protocol must also be provided in the manuscript.

Flow Cytometry

Plots

Confirm that:

- [x] The axis labels state the marker and fluorochrome used (e.g. CD4-FITC).
- [x] The axis scales are clearly visible. Include numbers along axes only for bottom left plot of group (a 'group' is an analysis of identical markers).
- [x] All plots are contour plots with outliers or pseudocolor plots.
- [x] A numerical value for number of cells or percentage (with statistics) is provided.

Methodology

Sample preparation	The spleen cells were stained for 30 minutes with fluorescent anti-mouse/human antibody on ice. Cells were washed in PBS 1% BSA once, and cells were stained with the live/dead stain.
Instrument	ImageStream mkII system (Merk Serono Co., Ltd., Darmstadt, Germany) was used for flow cytometry data collection.
Software	FlowJo Version V10 was used for data analysis.
Cell population abundance	At least 10,000 cells were analyzed for each condition.
Gating strategy	Cells were gated first by FSC/SSC, and then for Live/Dead negative (live cells).

- [x] Tick this box to confirm that a figure exemplifying the gating strategy is provided in the Supplementary Information.
